# Gamma frequency connectivity in frontostriatal networks associated with social preference is reduced with traumatic brain injury

**DOI:** 10.1162/netn_a_00416

**Published:** 2024-12-10

**Authors:** Morteza Salimi, Tianzhi Tang, Milad Nazari, Jyoti Mishra, Houtan Totonchi Afshar, Miranda Francoeur Koloski, Dhakshin S. Ramanathan

**Affiliations:** Mental Health Service, VA San Diego Healthcare System, La Jolla, CA, 92161, USA; Department of Psychiatry, UC San Diego, La Jolla, CA, 92093, USA; Department of Molecular Biology and Genetics, Aarhus University, Aarhus, Denmark; DANDRITE, The Danish Research Institute of Translational Neuroscience, Aarhus, Denmark; Center for Protein in Memory-PROMEMO, Danish National Research Foundation, Aarhus, Denmark; Center of Excellence for Stress and Mental Health, VA San Diego Healthcare System, La Jolla, CA, 92161, USA; Mental Health Care Line, VA San Diego Healthcare System, La Jolla, CA, 92161, USA

**Keywords:** Traumatic brain injury, Gamma frequency, Fronto-striatal networks, Social preference

## Abstract

Among the myriad of complications associated with traumatic brain injury (TBI), impairments in social behaviors and cognition have emerged as a significant area of concern. Animal models of social behavior are necessary to explore the underlying brain mechanisms contributing to chronic social impairments following brain injury. Here, we utilize large-scale brain recordings of local field potentials to identify neural signatures linked with social preference deficits following frontal brain injury. We used a controlled cortical impact model of TBI to create a severe bilateral injury centered on the prefrontal cortex. Behavior (social preference and locomotion) and brain activity (power and coherence) during a three-chamber social preference task were compared between sham and injured animals. Sham rats preferred to spend time with a social conspecific over an inanimate object. An analysis of local field oscillations showed that social preference was associated with a significant increase in coherence in gamma frequency band across widespread brain regions in these animals. Animals with a frontal TBI showed a significant reduction in this social preference, visiting an inanimate object more frequently and for more time. Reflecting these changes in social behavior, these animals also showed a significant reduction in gamma frequency (25–60 Hz) coherence associated with social preference.

## Author Summary

Our results indicate that disturbance in the gamma band network is associated with reduced social preference following frontal TBI. Importantly, the deficits we observed are network-wide, extending far beyond the focal site of injury. Our study identifies a potential neural signature of social preference that could be manipulated to improve social behaviors and may have translational relevance.

## INTRODUCTION

Traumatic brain injury (TBI) can lead to long-term impairments in cognitive, sensory, motor, and emotional domains that substantially affect everyday life (Mckee & Daneshvar, 2015). Impairments in social and emotional behaviors, including problems with communication, social cognition, and emotional regulation, are often long-lasting and associated with high levels of functional disability and depression (Barrash, Tranel, & Anderson, 2000; Benedictus, Spikman, & van der Naalt, 2010; Risbrough, Vaughn, & Friend, 2022; Shaver et al., 2019; Stein et al., 2019). Existing treatments for social deficits include the use of behavioral therapies, social enrichment, and pharmacological treatment (Baguley, Cooper, & Felmingham, 2006; Barrash et al., 2000; Ryan et al., 2016; Shaver et al., 2019). Although these techniques show efficacy, impairments persist for a large population (Bozkurt, Lannin, Mychasiuk, & Semple, 2023), suggesting the importance of better understanding the neural changes associated with these social deficits.

Preclinical animal models are an important tool toward understanding how acquired brain injuries contribute to social deficits but have been greatly understudied compared with cognitive or sensorimotor impairments. Many aspects of social behavior in humans rely on complex interpersonal dynamics, language, or other processes that are difficult to model in rodents. However, one aspect of social behavior that can be studied reliably in animal models is social preference: the tendency to favor interactions with conspecifics over inanimate objects (Bozkurt et al., 2023; Crawley, 2004; Fortier, Meisner, Nair, & Chang, 2022). When given the choice to explore a conspecific or a nonsocial stimulus (empty cage/object), rodents spend significantly more time interacting with the conspecific (Crawley, 2004). Prior work (using pediatric models and various approaches to deliver a brain injury) has shown that TBI can result in a reduction in social preference in rodents (Bondi et al., 2015; Ryan et al., 2016). However, there have been few studies that have studied the associated physiological changes.

Prior works on the neural circuits supporting social preference in humans and animals have focused on reward and decision-related circuitry. Thus, the prefrontal cortex (decision-making), ventral striatum/nucleus accumbens (positive approach toward social stimuli), and limbic regions like amygdala (observational fear, social preference, and reward) are all implicated (Allsop et al., 2018; Fernández, Mollinedo-Gajate, & Peñagarikano, 2018; Fortier et al., 2022; Manduca et al., 2016; Tremblay, Sharika, & Platt, 2017). The prefrontal cortex and its extended network are particularly susceptible to disruption after a brain injury (Warren et al., 2014), but the underlying mechanism of how damage to these pathways contributes to social impairments remains unknown. In rodents, studies have demonstrated that TBI leads to significant reductions in social preference, paralleling human social deficits. Specifically, previous works have shown that TBI in rodents impairs social preference, as evidenced by reduced interaction with conspecifics compared with inanimate objects (Bondi et al., 2015; Ryan et al., 2016).

To better uncover the neural networks contributing to social impairments after TBI, we wanted to use an approach of studying distributed brain networks. One approach for studying network-wide activity is through the measurement of neural oscillations (intracranially, as local field potentials, LFP) or extracranially, as electroencephalography or magnetoencephalography, providing translatability across species (Buzsáki & Watson, 2012; Masimore, Kakalios, & Redish, 2004. These oscillations are classically studied within specific frequency bands (delta, 1–4 Hz; theta, 4–8 Hz; alpha, 8–15 Hz; beta, 15–30 Hz; gamma, >30 Hz) that have been previously linked with different aspects of behavior and can predict disease states or response to treatment (Buzsáki & Watson, 2012; Masimore et al., 2004). We have previously used a novel recording technique to capture “brain-wide” LFP from 32 regions simultaneously and have identified signatures of sensory response mapping, inhibition, and reward expectation (Francoeur et al., 2021). Here, we employ this technique to capture brain signatures associated with social stimuli and investigate how frontal TBI impacts neural networks supporting social preferences.

Prior works in rodents have shown elevated gamma band (30–90 Hz) activity in the nucleus accumbens and prefrontal cortex during social behavior (Aguilar et al., 2021; Manduca et al., 2016). Stimulating cortical neurons at gamma frequencies (40 Hz) improved social deficits in a rodent autism model (where social interactions are impaired) (Cao et al., 2018). By employing advanced recording techniques to measure brain-wide neural oscillations, we aim to bridge the gap between rodent models and human studies, providing insights into how TBI affects distributed brain networks involved in social behavior. Moreover, our use of the controlled cortical impact (CCI) model of TBI in rats allows us to study severe frontal injuries that mimic concussion, contusion, and hemorrhage in humans, thereby enhancing the translatability of our findings and offering a basis for potential therapeutic approaches.

## RESULTS

The results are based on behavior and LFP recordings from 21 male Long-Evans rats from a single session of the three-chamber sociability test. Nine rats (control) underwent sham surgery, and 12 rats (TBI) received a bifrontal CCI injury. The dataset was part of a larger behavioral battery conducted up to 12 weeks post-TBI (results from probabilistic reversal learning task recently published; Koloski et al., 2024). The results below are from a three-chamber social preference task collected at 2 weeks post-TBI, a time after the initial neuroinflammation, hemorrhage, and axonal shearing, but before a chronic stage of recovery (Feeney, Boyeson, Linn, Murray, & Dail, 1981; Mao et al., 2020; Pearn et al., 2017, p. 1981). Further details are provided in the Materials and Methods section.

### Frontal TBI Decreases Preference for Social Stimulus

**Figure 1.**
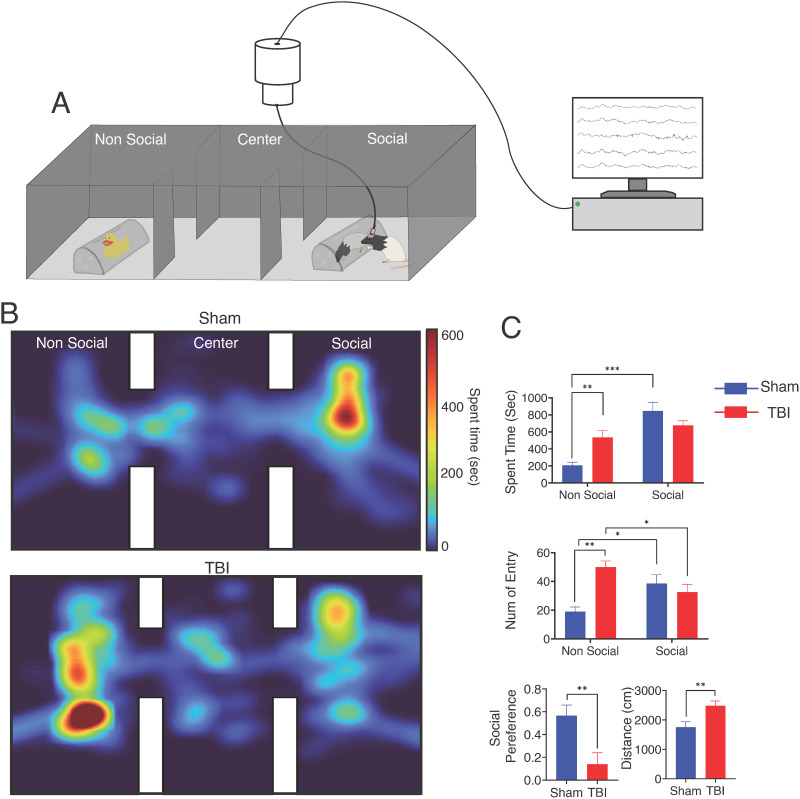
Behavioral assessments. (A) Schematic representation of the three-chamber social performance maze. One chamber contained a social stimulus (rat) and the other a nonsocial object (rubber duck). (B) Representative spatial map from a single session of one sham (top) and one TBI (bottom) animal in the three-chamber sociability chamber. The heat map indicates time spent (s) created by tracing the subject’s location in each frame. The size of each voxel was set to 10 mm × 10 mm, and the image was smoothed with a Gaussian filter. In this example, the sham rat spends more time in the social chamber and the TBI rat spends more time in the nonsocial chamber. (C) We quantified the total time spent in each chamber, the number of entries into each chamber, and the overall social preference score and locomotion. Only sham rats (blue) show a difference in time spent (s) between chambers (*p* < 0.001). TBI rats (red) spent more time in the nonsocial chamber than sham rats (*p* = 0.002). TBI rats also had more entries in the nonsocial chamber than sham rats (*p* < 0.001) and showed a lower social preference score (*p* = 0.007). Locomotion (distance traveled) was greater in the TBI group (*p* = 0.008). Mean and *SEM* are shown for each outcome variable. *p < 0.05, **p < 0.01, and ***p < 0.001.

On the three-chamber social task, we first examined how social behavior was different between rats with a frontal TBI and sham rats. In this task, subjects could choose between a social chamber with a conspecific or a nonsocial object (Figure 1A). We recorded their LFP signal as rats freely explore in these chambers (Figure 1A). Figure 1B represents an example session of spent time in the three-chamber maze for a sham and TBI rat (Figure 1B). Primary outcome measures including the time spent in each chamber and the number of entries were analyzed using a mixed-effects model with a between-subjects factor of group (TBI vs. sham injury) and a within-subjects factor of the chamber (social vs. nonsocial). First, measuring the time spent within each chamber, we found a significant main effect of the chamber (*F*_(1,19)_ = 18.75, *p* < 0.001) and a group × chamber interaction (*F*_(1,19)_ = 7.58, *p* = 0.01). Post hoc *t* tests (Sidak’s multiple comparisons) run separately for each chamber, which showed that animals with a TBI spent significantly more time in the nonsocial chamber compared with sham animals (TBI = 534.196 ± 79 s; sham = 206.7 ± 38.04 s, *p* = 0.003). Post hoc comparisons run separately in each group showed that sham animals spent significantly more time in the social chamber than the nonsocial chamber (*p* = 0.0003), while TBI animals did not spend a significantly different time between chambers (*p* = 0.42) (Figure 1B–C). Similarly, on analyzing the number of entries into the chambers, we observed a significant main effect of group (*F*_(1,19)_ = 5.99, *p* = 0.02) and a significant group × chamber interaction (*F*_(1,19)_ = 14.13, *p* = 0.0013). Post hoc *t* tests (Sidak’s multiple comparisons) show that the significant interaction was driven by more entries into the nonsocial chamber in TBI rats compared with the sham animals (*p* < 0.001). A within-group analysis indicated that sham animals more frequently entered the social chamber compared with the nonsocial chamber (*p* = 0.03), while TBI rats entered the nonsocial chamber more (*p* = 0.02) (Figure 1C).

Next, we calculated the social preference index (the proportion of time animals spend in the social chamber divided by the total time spent in both chambers) as an outcome variable. Social preference was significantly different between groups (*t*_(19)_ = 3.2, *p* = 0.007). TBI animals had a lower social preference score (0.14 ± 0.1) than sham animals (0.56 ± 0.09) (Figure 1C).

Finally, we analyzed the locomotor activity between groups with an independent samples *t* test. Rats with TBI traveled a greater distance (2,481 ± 162.8 cm) than sham animals (1,751 ± 185.3 cm) (*t*_(19)_ = 2.59, *p* = 0.008) (Figure 1C). In summary, the rats with frontal TBI spend less time and make fewer entries in the social stimulus chamber even while having overall more locomotor activity, resulting in reduced social preference compared with sham animals.

### TBI Reduced Power Spectra Activity During the Three-Chamber Social Task

**Figure 2.**
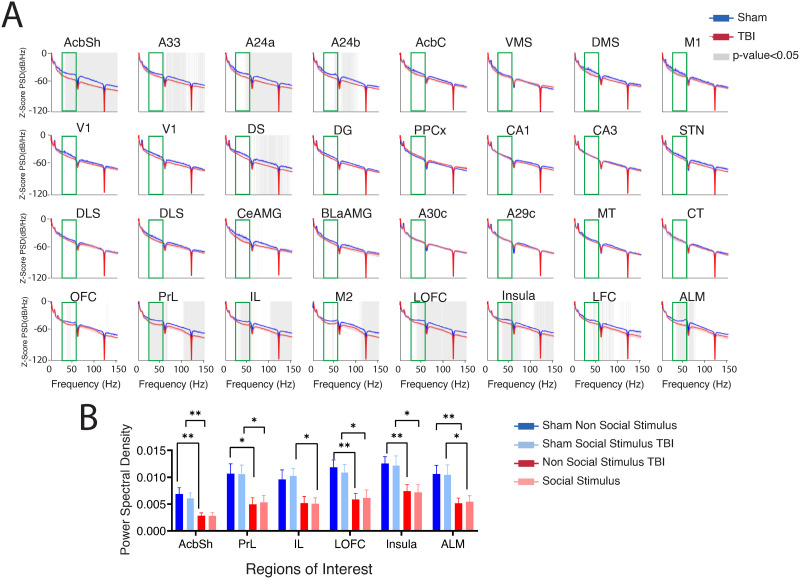
PSD. (A) Mean spectral power for sham (blue) and TBI (red) in all 32 regions when exploring in the social chamber. Gray transparent areas in the plots display statistically significant differences between sham and TBI (*p* < 0.05). The green rectangle indicates the frequency of interest 25–60 (Hz) analyzed by paired-sample *t* tests. Error bars represent *SEM*. TBI reduced the power of the signal in frontal areas and ventral striatum at gamma frequency bands, particularly 25–60 Hz. (B) Statistical analysis for PSD at 25–60 Hz in the regions of interest with the most significant between-group differences. Data were analyzed using ANOVA followed by Sidak’s correction for multiple comparisons, mean ± *SEM*, *p < 0.05 and **p < 0.01. TBI, traumatic brain injury; M2, motor area 2; Prl, prelimbic cortex; IL, infralimbic cortex; VO, ventral orbitofrontal cortex; M1, motor area 1; LFC, lateral frontal cortex; AI, anterior insula; LO, lateral orbitofrontal cortex; DMS, dorsomedial striatum; VMS, ventromedial striatum; AcbC, nucleus accumbens core; AcbSh, nucleus accumbens shell; MT, medial thalamus; CT, central thalamus; DLS, dorsolateral subiculum; CeAMG, central amygdala; BLaAMG, basolateral amygdala; STN, subthalamic nucleus; CA3, cornu ammonis 3 hippocampus; CA1, cornu ammonis 1 hippocampus; PPx, posterior parietal cortex; DG, dentate gyrus; V1, visual cortex 1.

To explore patterns of activity-related social behavior, we first explored data within the sham animals to see if power across brain regions and frequencies differentiated social versus nonsocial preference. In this exploratory analysis, we analyzed power among all 32 electrodes across all frequencies. We did not observe meaningful significant differences in sham animals as a function of the chamber (Supporting Information Figure S1). Moreover, no significant changes were found in the TBI animals between the two chambers (Supporting Information Figure S2). Because of this, we next focused on exploring differences between groups solely within the social chamber (Figure 2A). Comparing power spectra activity between groups during social stimulus interaction, we broadly found diminished power in TBI rats (Figure 2A). Loss of power in TBI rats was observed within frontal and reward-related subcortical brain regions and tended to show the greatest number of significant differences within higher frequency (>25 Hz) gamma bands (Figure 2). These significant differences did not survive false discovery rate (FDR) correction (across regions/frequency bands), so we are showing that these as uncorrected.

Among all the frequency bands examined, the gamma band exhibited the most significant changes in our study. This observation aligns with previous research indicating that gamma oscillations are particularly sensitive to alterations in neural synchrony and connectivity, which are often disrupted in social cognitive disorders. We identified six brain regions that exhibited the largest number of significant between-group differences (independent *t* tests) in the social chamber for further analysis in gamma frequencies (25–60 Hz). The selection of the gamma frequency band was deliberate and grounded in previous research findings. This frequency band has been shown to play a crucial role in the synchronization of neural activity across different brain regions, which is essential for social cognition and the integration of social information, and deficits in gamma oscillatory activity have been linked to impairments in social functioning, underscoring the relevance of this frequency band in understanding social deficits (Aguilar et al., 2021).

We used a mixed-model analysis of variance (ANOVA) to examine gamma power differences between groups (2) × electrodes (6) × chamber (2). Consistent with our prior observations, we found a significant group effect (*F*_(1,19)_ = 10.67, *p* = 0.004) and a significant electrode effect (*F*_(3.24,61.6)_ = 9.74, *p* < 0.001; Figure 2B), but no group × electrodes interaction (*F*_(3.24,66.4)_ = 0.527, *p* = 0.67) nor any interactions with chamber (*F*_(1,19)_ = 0.11, *p* = 0.73). Our post hoc analysis indicated significant main effects of injury in the nucleus accumbens shell (*p* = 0.008 in social and *p* = 0.003 in the nonsocial chamber), in the prelimbic cortex (*p* = 0.018 in social and *p* = 0.015 in the nonsocial chamber), in the infralimbic cortex (*p* = 0.048 in social, but not significant in the nonsocial chamber, *p* = 0.074), in the lateral orbitofrontal cortex (*p* = 0.037 in social and *p* = 0.003 in the nonsocial chamber), in the anterior insula (*p* = 0.04 in social and *p* = 0.01 in the nonsocial chamber), and in the anterolateral motor cortex (*p* = 0.022 in social and *p* = 0.007 in the nonsocial chamber). Thus, in summary, TBI reduced gamma power across multiple electrodes spanning the cortex and striatum; but this reduction occurred in both social and nonsocial chambers and, thus, does not readily explain the social preference deficits observed behaviorally.

### TBI Disrupts Functional Connectivity Relevant to Social Interaction at Gamma Frequencies

**Figure 3.**
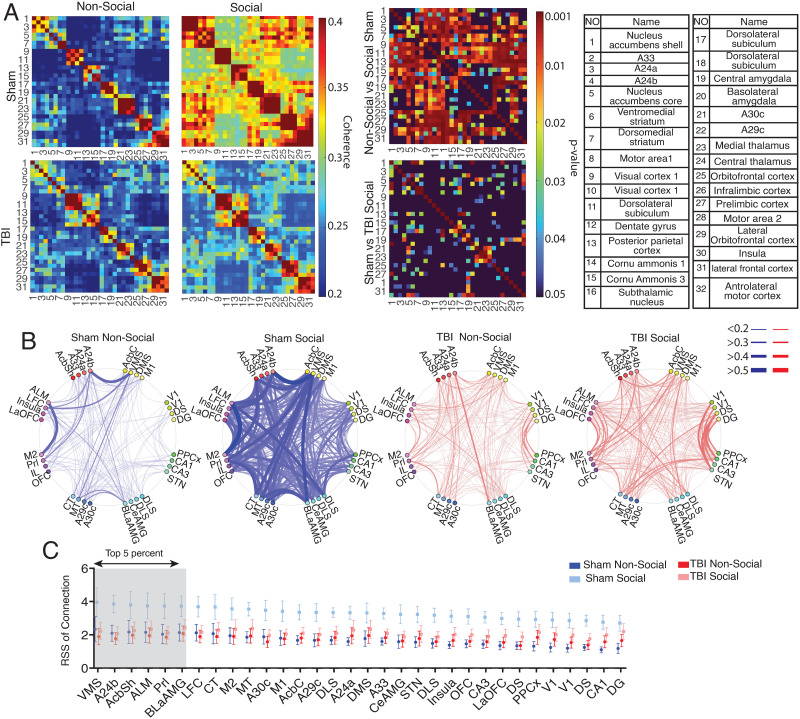
Map of coherence. (A) A 2D histogram of coherence between 32 brain regions at 25- to 60-Hz frequency band. Each cell represents the value of coherence between two brain regions marked by electrode number (table). Blue to dark red colors indicate increased coherence from low to high. The right matrices show the significance values (uncorrected *p* value) of coherence for each pairwise connection. Blue to dark red colors indicate increased significance from low to high. (B) The connectogram is plotted based on values of the coherence between pairwise regions for sham (blue) and TBI (red) in nonsocial (light) and social (dark) chambers. Electrodes are organized by their cannula bundles, and connections between electrodes on the same bundle are not analyzed. The thickness of the lines denotes the value of the coherence. (C) Thirty-two brain regions were sorted based on the RSS of coherence for sham animals in the social chamber. Two-way ANOVA analysis indicated a significant group × chamber interaction (*F*_(1,19)_ = 6.39, *p* = 0.02), but no effect of electrode (*F*_(1,19)_ = 2.45, *p* = 0.07). Shaded areas represent the highest RSS values, based on the sham rats social chamber activity, and were selected for further coherence analyses. M2, motor area 2; Prl, prelimbic cortex; IL, infralimbic cortex; VO, ventral orbitofrontal cortex; M1, motor area 1; LFC, lateral frontal cortex; AI, anterior insula; LO, lateral orbitofrontal cortex; DMS, dorsomedial striatum; VMS, ventromedial striatum; AcbC, nucleus accumbens core; AcbSh, nucleus accumbens shell; MT, medial thalamus; CT, central thalamus; DLS, dorsolateral subiculum; CeAMG, central amygdala; BLaAMG, basolateral amygdala; STN, subthalamic nucleus; CA3, cornu ammonis 3 hippocampus; CA1, cornu ammonis 1 hippocampus; PPx, posterior parietal cortex; DG, dentate gyrus; V1, visual cortex 1.

As a global reduction in gamma power in both social and nonsocial chambers may not fully explain behavioral social preference deficits in the TBI group, we next explored whether network coherence may be a more sensitive measure to explain network-wide changes modulating social behavior. Phase coherence refers to phase-synchronized neural oscillatory activity between distinct electrodes/brain regions and has been used as a potential proxy for inter-areal communication and functional connectivity (Fries, 2015). We measured the differences in gamma coherence between electrodes as a function of chamber (social vs. nonsocial) or group (TBI vs. sham). As before, we began our analysis by exploring whether there was a significant difference between the social and nonsocial chambers in sham animals (i.e., is there a neural signature associated with social preference). We found a significant increase in gamma coherence for the social compared with the nonsocial chamber in the sham across many brain regions (Figure 3A; Supporting Information Figure S3). In sham rats, 327 significant coherence values were greater in the social chamber than the nonsocial chamber (Figure 3A, showing data without FDR correction). However, in TBI rats, there were no significant coherence pairs that were greater in social than nonsocial (Supporting Information Figure S4). Comparing groups, sham rats had greater coherence values than TBI rats at 98 electrode pairs during social interaction (Figure 3A; Supporting Information Figure S5).

Given the large number of pairwise tests in the above analysis, we took an additional step to reduce the total number of analyses performed when analyzing the effects of injury. To do this, we first converted the matrix of coherence values into a connectogram—a pictorial representation of coherence strengths (Figure 3B). For this analysis, we ignored electrodes that were implanted together on the same cannula to minimize the chance of spurious/artifactual correlations driven by proximity. For each brain region, we next calculated the root square summation (RSS) from the connectogram (Figure 3C; plotting mean/standard error of mean for each electrode). The RSS is a combination of the number of connections and the strength of each connection and, thus, provides a single number for estimating the weighted connectivity from one region to all other regions. We first analyzed using a group (sham vs. TBI) × chamber (social vs. non-social) × electrode model. We found a significant group × chamber interaction (*F*_(1,19)_ = 6.39, *p* = 0.02) and chamber effect (*F*_(1,19)_ = 15.22, *p* < 0.001). We did not observe a main effect of an electrode (*F*_(72,562.7)_ = 2.45, *p* = 0.07) nor any significant group × electrode interactions (*F*_(1,19)_ = 1.34, *p* = 0.26), meaning that the differences we observe are widespread throughout most brain regions (Figure 3C).

To further explore between-group differences in connectivity during social interactions, we next probed pairwise interactions. To select for specific pairwise interactions in a data-driven and unbiased manner, we sorted electrodes in a descending order of RSS scores from sham animals in the social chamber (Figure 3C). The following six brain regions were in the top fifth percent of RSS values based on sham social data: ventromedial striatum (VMS), cingulate cortex (A24b), nucleus accumbens shell (Acb Sh), anterolateral motor cortex (ALM), prelimbic cortex (Prl), and basolateral amygdala (BLA). By sorting/selecting electrodes based solely on the sham animals, we optimized our selection for brain regions, showing the greatest connectivity strength during the social preference without optimizing/selecting for regions that would be most likely impaired by the TBI, that is, selection was data-driven and not biased toward finding an effect of the injury.

**Figure 4.**
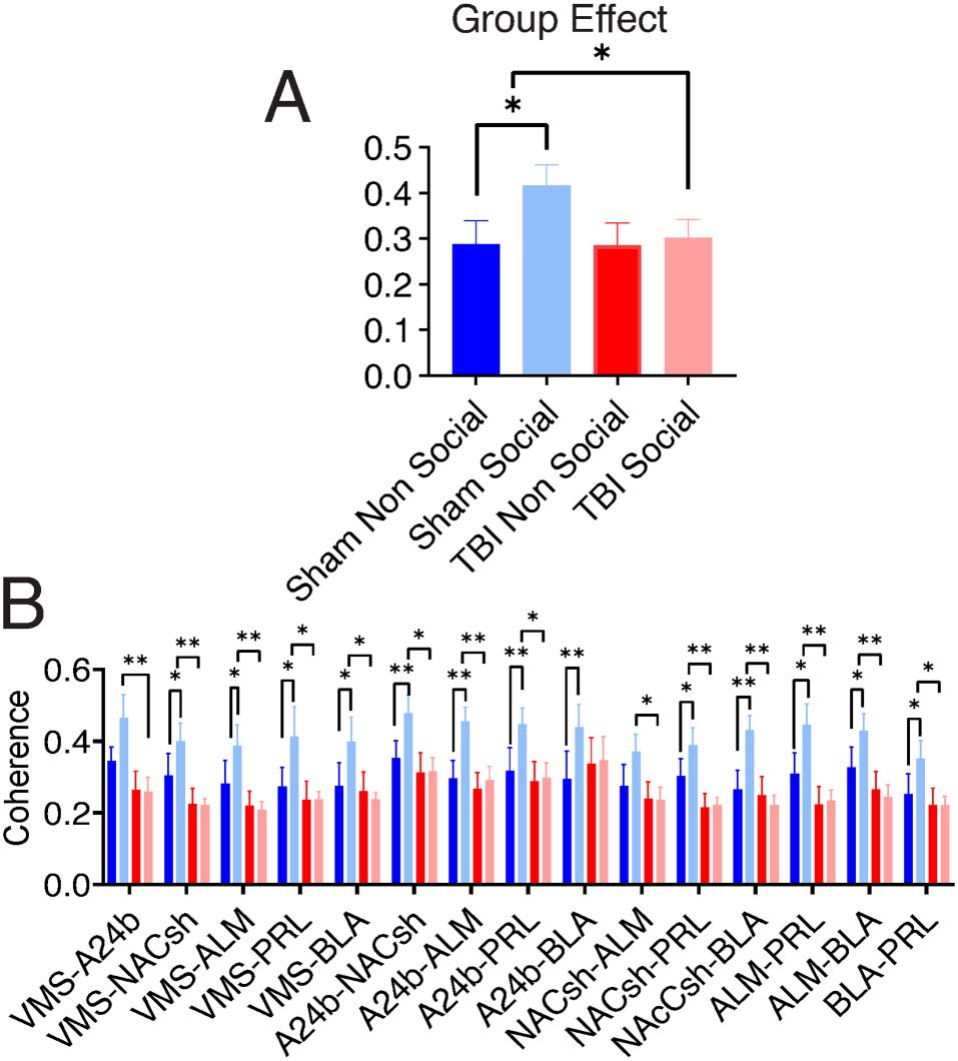
Coherence comparison in regions of interest. (A) The average across electrode pairs (no significant effects of electrode) shows the between-group (TBI vs. sham) and within-group (nonsocial vs. social) comparisons. Bar graphs denote mean ± *SEM* for all paired electrodes. (B) Statistical comparison for pairwise regions with higher RSS. Data were analyzed using ANOVA followed by Sidak’s post hoc correction for comparisons, *p < 0.05 and **p < 0.01. VMS, ventromedial striatum; ALM, anterolateral motor cortex; Prl, prelimbic cortex; IL, infralimbic; AcbSh, nucleus accumbens shell; BLaAMG, basolateral amygdala.

Next, using the top fifth percentile as defined by sham, social RSS values, we measured coherence between six electrodes. First, we looked at the average group (TBI vs. sham) and chamber (social vs. nonsocial) differences in coherence for all six electrodes of interest. Our analyses indicated a significant group × chamber interaction (*F*_(1,19)_ = 8.97, *p* = 0.007), significant chamber effect (*F*_(1,19)_ = 8.14, *p* = 0.010) and group effect (*F*_(1,19)_ = 4.77, *p* = 0.042; Figure 4A). In sham animals, functional connectivity increased when exploring the social stimulus compared with the nonsocial stimulus. This was unique to sham animals. TBI animals did not show a difference in functional connectivity between chambers. Moreover, group effect analysis indicated connectivity between brain circuits that was significantly greater in the sham animals for the social versus nonsocial chamber (0.416 ± 0.045 social; 0.288 ± 0.051 nonsocial), and this difference was greater in the sham compared with TBI animals (0.318 ± 0.039 social; 0.284 ± 0.048 nonsocial). Next, we ran planned comparisons (group × chamber ANOVA) for each pair of the six electrodes in the top fifth percentile (15 comparisons in total) followed by Sidak’s multiple comparison correction (Figure 4B, Table 1). Our post hoc within-group analysis (Table 1) indicated 27 significant differences between electrode pairs. Together, in the circuitry between the specified brain regions, the coherence of brain oscillations at the gamma frequency band was higher in sham rats when they were exploring social stimulus compared with nonsocial stimulus. In contrast, TBI led to a condition where coherence remained unchanged for exploring social versus nonsocial stimuli, but it was reduced compared with the sham group.

**Table 1.** Pairwise coherence values showing significant group and chamber effects in bold

	Mean ± SEM				Statistical comparison			
	Sham		TBI		Chamber effect		Group effect	
	Nonsocial	Social	Nonsocial	Social	Sham	TBI	Nonsocial	Social
VMS-A24b	0.33 ± 0.04	0.46 ± 0.06	0.26 ± 0.05	0.24 ± 0.02	*t*_(8)_ = 1.88 *p* = 0.08	*t*_(11)_ = 0.32 *p* = 0.75	*t*_(19)_ = 0.97 *p* = 0.34	***t*_(19)_ = 3.41 *p* = 0.003**
VMS-NACsh	0.30 ± 0.06	0.39 ± 0.05	0.22 ± 0.04	0.22 ± 0.01	***t*_(8)_ = 3.76 *p* = 0.04**	*t*_(11)_ = 0.07 *p* = 0.93	*t*_(19)_ = 1.08 *p* = 0.29	***t*_(19)_ = 3.67 *p* = 0.002**
VMS-ALM	0.28 ± 0.06	0.38 ± 0.05	0.21 ± 0.04	0.20 ± 0.02	***t*_(8)_ = 4.6 *p* = 0.04**	*t*_(11)_ = 0.22 *p* = 0.82	*t*_(19)_ = 0.82 *p* = 0.41	***t*_(19)_ = 3.12 *p* = 0.006**
VMS-PLC	0.27 ± 0.05	0.41 ± 0.08	0.23 ± 0.05	0.23 ± 0.02	***t*_(8)_ = 3.78 *p* = 0.03**	*t*_(11)_ = 0.05 *p* = 0.95	*t*_(19)_ = 0.49 *p* = 0.62	***t*_(19)_ = 2.29 *p* = 0.03**
VMS-BLA	0.27 ± 0.06	0.39 ± 0.07	0.26 ± 0.05	0.23 ± 0.01	***t*_(8)_ = 3.74 *p* = 0.03**	*t*_(11)_ = 0.41 *p* = 0.68	*t*_(19)_ = 0.16 *p* = 0.87	***t*_(19)_ = 2.54 *p* = 0.02**
A24b-NACsh	0.35 ± 0.04	0.47 ± 0.04	0.31 ± 0.05	0.31 ± 0.03	***t*_(8)_ = 2.82 *p* = 0.005**	*t*_(11)_ = 0.8 *p* = 0.93	*t*_(19)_ = 0.52 *p* = 0.6	***t*_(19)_ = 2.26 *p* = 0.01**
A24b-ALM	0.29 ± 0.05	0.45 ± 0.03	0.26 ± 0.04	0.29 ± 0.03	***t*_(8)_ = 2.35 *p* = 0.005**	*t*_(11)_ = 0.81 *p* = 0.43	*t*_(19)_ = 0.42 *p* = 0.67	***t*_(19)_ = 2.94 *p* = 0.008**
A24b-PRL	0.31 ± 0.06	0.44 ± 0.04	0.28 ± 0.05	0.29 ± 0.04	***t*_(8)_ = 3.38 *p* = 0.006**	*t*_(11)_ = 0.23 *p* = 0.82	*t*_(19)_ = 0.33 *p* = 0.74	***t*_(19)_ = 2.33 *p* = 0.031**
A24b-BLA	0.29 ± 0.07	0.43 ± 0.06	0.33 ± 0.07	0.34 ± 0.06	***t*_(8)_ = 3.61 *p* = 0.001**	*t*_(11)_ = 0.29 *p* = 0.77	*t*_(19)_ = 0.39 *p* = 0.70	*t*_(19)_ = 0.99 *p* = 0.33
AcbSh-ALM	0.27 ± 0.05	0.37 ± 0.04	0.23 ± 0.04	0.23 ± 0.03	*t*_(8)_ = 3.85 *p* = 0.08	*t*_(11)_ = 0.07 *p* = 0.94	*t*_(19)_ = 0.49 *p* = 0.62	***t*_(19)_ = 2.30 *p* = 0.03**
Acbsh-PRL	0.30 ± 0.04	0.38 ± 0.04	0.21 ± 0.03	0.22 ± 0.02	***t*_(8)_ = 4.57 *p* = 0.04**	*t*_(11)_ = 0.21 *p* = 0.83	*t*_(19)_ = 1.44 *p* = 0.16	***t*_(19)_ = 3.46 *p* = 0.003**
Acbsh-BLA	0.26 ± 0.05	0.43 ± 0.03	0.24 ± 0.05	0.22 ± 0.02	***t*_(8)_ = 6.33 *p* = 0.004**	*t*_(11)_ = 0.5 *p* = 0.62	*t*_(19)_ = 0.18 *p* = 0.42	***t*_(19)_ = 3.45 *p* = 0.001**
ALM-PRL	0.34 ± 0.06	0.44 ± 0.05	0.22 ± 0.05	0.23 ± 0.02	***t*_(8)_ = 4.11 *p* = 0.05**	*t*_(11)_ = 0.21 *p* = 0.83	*t*_(19)_ = 1.46 *p* = 0.16	***t*_(19)_ = 3.41 *p* = 0.003**
ALM-BLA	0.32 ± 0.05	0.42 ± 0.05	0.26 ± 0.04	0.24 ± 0.03	***t*_(8)_ = 4.64 *p* = 0.027**	*t*_(11)_ = 0.46 *p* = 0.65	*t*_(19)_ = 0.81 *p* = 0.42	***t*_(19)_ = 3.29 *p* = 0.004**
BLA-PRL	0.25 ± 0.06	0.35 ± 0.05	0.22 ± 0.04	0.22 ± 0.02	***t*_(8)_ = 4.24 *p* = 0.047**	*t*_(11)_ = 0.02 *p* = 0.98	*t*_(19)_ = 0.40 *p* = 0.69	***t*_(19)_ = 2.54 *p* = 0.020**

## DISCUSSION

Our study contributes to an understanding of how neural networks support social interactions, and how these networks may be disturbed after frontal TBI. Impairments in social and emotional behaviors following TBI include apathy, inflexibility, and antisocial tendencies and represent a significant area of concern due to their impact on daily functioning (Barrash et al., 2000; Manduca et al., 2016; Risbrough et al., 2022; Ryan et al., 2016; Shaver et al., 2019; Tremblay et al., 2017). Yet, most preclinical TBI research focuses on cognitive and motor impairments and social preference deficits that are not as commonly studied. To our knowledge, this is the first study to examine oscillatory/physiological changes in conjunction with social preference deficits in experimental TBI. We made three main findings in this article. (a) We observed a reduction in social preference/social behaviors after a frontal brain injury. (b) In sham animals, we observed a widespread increase in gamma frequency coherence during social compared with nonsocial preference. These differences were not readily observable when looking solely at power. (b) Frontal TBI was associated with a specific reduction in the elevated gamma coherence associated with social preference observed in the sham animals. This network, while widespread, clearly implicates regions previously associated with social reward (ventral striatum and limbic).

Prior work has shown that experimental forms of TBI can result in various rodent social deficits (Shultz et al., 2020). Several prior studies have described reduced social interactions on a three-chamber test similar to what was used here using other forms of TBI (such as repetitive concussions; Klemenhagen, O’Brien, & Brody, 2013; Nolan et al., 2018; Shultz et al., 2020; Yu et al., 2017). Thus, our results are consistent with a larger body of work that shows frontal-acquired injuries can be accompanied by reduced social interactions. Importantly, none of that prior work interrogated physiological changes that could help to explain the social interaction deficits. Thus, our findings here complement and extend prior work in the field.

It is noteworthy that we did not observe any specific differences in power for the social versus nonsocial chambers in sham animals. Prior work in the field has suggested that social preference is linked with elevated gamma power, although many of these studies did not specifically contrast gamma power between chambers. For example, one key study (Aguilar et al., 2021) demonstrated that low gamma oscillations (30–50 Hz) were reduced in animals with a genetic knockout during social interaction, but they did not compare with changes in the object, so it is difficult to know if this reduction was specific to social preference (vs. just any form of exploratory behavior). A different study showed elevated firing of parvalbumin (PV) cells and elevated gamma power during social preference, but here, the comparison was to activity recorded in a chamber with nothing to explore, “neutral chamber” (Liu et al., 2020). The above papers both manipulated PV cells with expected results: Activating PV inhibitory neurons helped restore intact social interactions. Thus, our findings (lack of differential activity in social vs. novel object preference) may be different from prior reports due to differences in the experimental design of our task.

We did observe an overall reduction in gamma power in several brain regions following TBI that were unrelated to the social versus object preference (it was reduced in both chambers). Interestingly, these reductions in gamma power occurred both at the focal site of injury (dorsal prefrontal cortex) and regions distal to the area of impact (ventral striatum, amygdala), suggesting that the injury affects widespread networks. Gamma frequency activity in the prefrontal cortex may reflect microcircuit interactions between inhibitory (particularly PV) and excitatory glutamatergic projections (Sohal, Zhang, Yizhar, & Deisseroth, 2009). Excitatory/inhibitory interactions have been implicated in higher level cognition (Cho et al., 2020), and the balance may be critical for maintaining appropriate social behavior (Fernández et al., 2018). Gamma oscillations have also been shown to be synchronized across distant brain regions during cognition in both animals (Guan et al., 2022) and humans (Fries, Nikolić, & Singer, 2007; Herrmann & Demiralp, 2005), suggesting involvement in long-range networks. Prior work in preclinical models has shown that PV cells may be particularly susceptive to injury after TBI (Huusko & Pitkänen, 2014). Consistent with this, studies in humans have shown gamma band abnormalities following TBI, including reduced synchrony at gamma band frequencies (Guan et al., 2022) and abnormally elevated gamma band activity at rest (Huang, Zucca, Levy, & Page, 2020).

Sham animals displayed increased gamma coherence across a large number of brain regions in the social compared with the nonsocial chamber. We quantified and statistically analyzed this, using a measure of weighted connectivity (RSS). This provides a measure of the strength/number of connections between brain regions. During social preference, sham rats had stronger weighted connectivity (RSS values) during social versus object preference, and this difference was abolished in animals with a TBI. Sorting RSS values based on sham rats’ social chamber activity, we found the strongest weighted connectivity in the ventral striatum (VMS and nucleus accumbens shell), basolateral amygdala, and several prefrontal sites, notably A24b (part of the frontal orienting fields noted to be involved in attentional control), anterolateral motor cortex, and prelimbic cortex during social preferences. Weighted connectivity for many of these electrode pairs was significantly diminished in TBI animals in the social chamber. Thus, our results highlight gamma frequency deficits that encompass an extended corticostriatal limbic reward network that may explain social preference deficits observed in TBI animals.

Field potentials are complicated to interpret, as they can reflect both local and global processes and are further influenced by both neural and nonneural brain cells. Our study suggests some possible impairment in PV cell activity locally that may contribute to larger scale connectivity deficits during social behaviors. However, further work, measuring activity at the level of single units or in genetically determined subpopulations of neurons following TBI, will be an essential part of better understanding how these oscillatory deficits arose. TBI-induced neuroinflammation has been suggested as another potential mechanism underlying social decline. Neuroinflammation, characterized by the activation of immune cells in the brain, can disrupt neural communication and impair social behavior (Drieu et al., 2022; Li, Concepcion, Meng, & Zhang, 2017). Increased levels of proinflammatory cytokines, such as interleukin-6 (IL-6) and tumor necrosis factor-alpha, have been observed following TBI and are associated with social impairments (Oh et al., 2021). Our findings may be a result of the changes noted above, and further work will help to clarify the degree to which the network-level changes we observe are linked with neurotransmitter and/or inflammatory processes.

A strength of our study is the large-scale recording of multiple brain areas simultaneously. Most studies measuring neural activity during social behaviors are limited in the number of recording sites and rely on a prior hypothesis about areas of interest. Social interactions require integration of context and experience through multiple sensory modalities and, therefore, depend on a network of brain areas. Harnessing two-photon imaging to simultaneously measure activity from multiple brain areas, Kim et al. (2015) found increased c-fos expression (a marker of neural activity) in the olfactory bulb, hypothalamus, lateral septum, amygdala, nucleus accumbens, and prefrontal cortex during social interaction. Similarly, we consistently find widespread activation of the prefrontal cortex, ventral striatum, and amygdala during social stimulus interaction. Importantly, our results show that neurological processes are disrupted in extended networks, beyond the focal area of impact. These results align with previous studies that have highlighted the involvement of a network facilitating social interaction (Schwedt, 2019; Sharp, Scott, & Leech, 2014).

There are a few limitations with our current design. First, our results only include male rats. The preference for social interaction has been observed in male and female rodents, at juvenile and adult ages (Crawley, 2004). However, preclinical TBI data generally find worse behavioral outcomes in males dependent on factors such as injury model, severity, number of impacts, and sample size (Gupte, Brooks, Vukas, Pierce, & Harris, 2019; Rubin & Lipton, 2019), and therefore, sex differences require further investigation. Second, social testing was done in the light phase. Importantly, we do not think this will alter our findings: Although rats are naturally nocturnal animals, prior work has not suggested this to be a significant source of variability in social interaction tests (Yang, Weber, & Crawley, 2008). As mentioned above, LFPs are limited in what can be grasped, and thus, further work measuring activity at the level of single cells will be important to complement these studies. Last, although considered a classic tool for assessing rodent sociability, the three-chamber test is limited in its complexity (excludes the number, length, and quality of interactions), standardization between protocols, and ability to examine emotional state, and therefore, future directions will aim to apply this approach to different behavioral models (Yang et al., 2008).

In conclusion, decreased social preference observed in rats with frontal TBI was accompanied by gamma frequency power and connectivity deficits in widespread frontal-striatal brain regions. These findings suggest that TBI-induced alterations in gamma oscillations and network connectivity contribute to deficits in social preference. Future research should explore the underlying mechanisms, including neurotransmitter systems and neuroinflammation, to improve our understanding and develop potential interventions for individuals with TBI.

## MATERIALS AND METHODS

### Ethics Statement

All procedures were conducted in accordance with the Guide for the Care and Use of Laboratory Animals of the National Institutes of Health. The experimental protocol was approved by the San Diego VA Medical Center Institutional Animal Care and Use Committee (Protocol Number A17-014; A21-012).

### Animals

Male Long-Evans rats (Charles River, Wilmington, MA; *N* = 28) were used for this experiment. Rats were received at approximately 1 month old, weighing 150 g. All animals were pair-housed in standard rat cages (10 × 10.75 × 19.5 in Allentown, NJ, USA) before surgery and individually housed following surgery. Rats were kept on a standard light cycle (lights on at 6 AM/lights off at 6 PM) with free access to food. Water was restricted (2 hr of free access per day) to maintain motivation for water reward in operant conditioning tasks that were also performed in these animals (data not included in this manuscript). Water restriction began at the start of pretraining/habituation (~5 months old). Rats were weighed weekly and had free access to water on nonoperant training days. Three animals died as a result of TBI surgery complication, and five rats were excluded due to a lack of electrophysiology signal, resulting in 21 rats being used in analyses. The same rats were used for data reported by Koloski et al. (2024) investigating behavioral deficits on the probabilistic reversal learning task.

### Surgery

Animals underwent two surgeries: first for TBI-CCI or sham procedures (Day 0) and second for electrode implantation (Day 7) (Figure 4A). Rats were ~ 7 months old at the time of surgery. For all surgical procedures, the rat was anesthetized with isoflurane (2–4% in 0.5 L/min of oxygen) and placed in a stereotaxic frame with a heating pad (SomnoSuite, Kent Scientific, CT, USA; temperature adjusted to maintain a target body temperature of 37.5 °C). Animals received a single dose of atropine (0.05 mg/kg, subcutaneous (s.c.)) to diminish respiratory secretions during surgery, and 0.5–1 ml of 0.9% sterile saline (s.c.). The area of the incision was cleaned with 70% ethanol and iodine solution, and injections of lidocaine (max of 0.2 ml) were given subdermal for local anesthetic. A 2.0-cm incision was made along the midline, and the tissue was cleared to expose the skull.

#### CCI-TBI.

Twelve rats received CCI surgery, and nine rats underwent sham procedures. Following previous protocols (Koloski et al., 2024; Ma, Aravind, Pfister, Chandra, & Haorah, 2019; Vonder Haar et al., 2017), TBI rats received a 6.0-mm-diameter circular craniotomy centered on the prefrontal cortex (anterior posterior +3.0, medial lateral +0.0 from bregma) using a microdrill. A stainless-steel circular impactor tip (5.0 mm diameter) was positioned over the craniotomy and an electromagnetically controlled cortical impactor (Leica Biosystems, Buffalo Grove, IL) was used to induce a severe injury (2.5 mm of depth; 3 m/s of velocity; 500 ms of dwell) (Figure 1B). Bleeding was controlled, craniotomy was sealed, and incision was sutured. Sham rats underwent a similar surgical procedure (pre-op medication, midline incision, expose skull surface, suture incision) but did not receive a craniotomy or injury. Time under anesthesia was matched for sham and TBI rats. Rats were given a single dose (1 mg/kg) of buprenorphine SR for pain management and placed in a heated recovery chamber until they regained consciousness. Rats received sulfamethoxazole and trimethoprim in their drinking water (60 mg/kg for 5 days) to prevent infections.

#### Electrophysiology implants.

**Figure 5.**
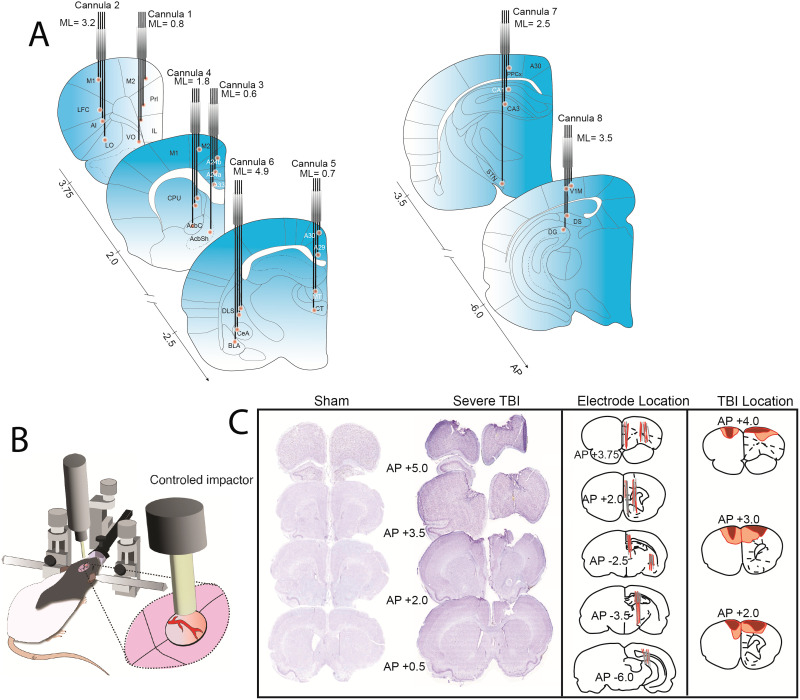
Protocol of the study. (A) Schematic representation of 32 electrode locations using eight cannulas. Each cannula contains four microwires, each targeting a unique DV location (8 cannulas × 4 wires = 32 sites). The coronal sections show the target location for each microwire. Coordinates are based on bregma. (B) Schematic representation for TBI model induction using CCI. (C) Histological confirmation for TBI injury and electrode placement. Nissl (thionine)-stained coronal sections are shown for example sham and TBI subjects at AP +5.0, 3.5, 2.0, and 0.5 relative to bregma. TBI produces bilateral lesions and damage to cell bodies. Although the injury is centered on the midline during surgery, the resulting damage may not be perfectly symmetrical at chronic time points. The schematic representation on the right illustrates the distribution of injury, with the dark red color indicating the minimum severity and light red color indicating the maximum severity. M2, motor area 2; Prl, prelimbic cortex; IL, infralimbic cortex; VO, ventral orbitofrontal cortex; M1, motor area 1; LFC, lateral frontal cortex; AI, anterior insula; LO, lateral orbitofrontal cortex; DMS, dorsomedial striatum; VMS, ventromedial striatum; AcbC, nucleus accumbens core; AcbSh, nucleus accumbens shell; MT, medial thalamus; CT, central thalamus; DLS, dorsolateral subiculum; CeAMG, central amygdala; BLaAMG, basolateral amygdala; STN, subthalamic nucleus; CA3, cornu ammonis 3 hippocampus; CA1, cornu ammonis 1 hippocampus; PPx, posterior parietal cortex; DG, dentate gyrus; V1, visual cortex 1.

After recovering for 1 week, rats underwent a second surgery to implant the 32 LFP microwires. The probe fabrication and surgical procedures have been previously described in detail (*DANDI Archive*, n.d.; Francoeur et al., 2021). Surgery starts as stated above with isoflurane anesthesia, pre-op medication (atropine and saline), and midline incision. After clearing the skull, eight holes (0.9 mm diameter) were drilled to implant microwires (50-μm tungsten; California Fine Wire, Grover Beach, CA) at predetermined stereotactic locations. Each hole drilled was for a cannula containing four microwires precut to individual lengths based on the desired dorsal ventral measurement of the target brain region (Figure 5A). An additional hole was drilled above the cerebellum for the ground wire soldered to an anchor screw. Several more holes were drilled for anchor screws (five to eight) at the skull periphery. Each cannula was lowered to the desired depth, secured with Metabond (Parkell, NY, USA), pinned to an Omnetics Electrode Interface Board (Neuralynx, MT, USA), and encased in dental cement (Stoelting, IL, USA). After surgery, the skin was sutured, and rats were given a single dose (1 mg/kg) of buprenorphine SR for pain management. Rats recovered from surgery in a heated chamber and received sulfamethoxazole and trimethoprim in their drinking water (60 mg/kg for 5 days).

### Behavioral Apparatus

We used a standard three-chamber maze, 80 × 40 × 40 cm in size (Maze Engineers, Skokie, IL, USA). The three-chamber maze had a clear polycarbonate bottom, black sides and inserts, and an open top to allow electrophysiology cables to move freely. The chambers were separated by dividers. We removed the doors between chambers to prevent obstruction of cables. Two round clear acrylic cages (15 cm diameter × 30 cm height; 1-cm spacing between slits) were placed in the lateral chambers to house a rat (social stimuli) or object (nonsocial stimuli). The central chamber was left empty. The sociability chamber was housed on a shelf in a custom-made box (122 cm × 92 × 122 cm), with houselights on to facilitate video tracking. A video camera (Arducam Wide Angle, Amazon) recorded activity from the bottom of the sociability chamber. The video was recorded at 20 fps and saved as an .mp4 file.

Electrophysiology data were recorded using Tucker-Davis Technologies (TDT; FL, USA) system. The camera used to record behavior was connected and stored within the TDT system, allowing for time stamps from each frame to be recoded and synced with electrophysiological recordings. A 32-channel RHD headstage (Intan Technologies, CA, USA) was coupled to a motorized commutator (ACO32, TDT) with an Serial Peripheral Interface cable. The commutator was connected to a PZ5 NeuroDigitizer and RZ2 BioAmp Processor (TDT). LFPs were processed using Synapse software (TDT) and recorded at a sampling rate of 6 kHz, 1,000-Hz low-pass filter, 0.1-Hz high-pass filter, and 60-Hz notch filter. Raw data are stored as a .tev file.

### Behavioral Testing

The data reported here were part of a larger cognitive and social battery study examining behavior at multiple time points up to 12 weeks following TBI. Subjects (sham and TBI) were evaluated in a three-chamber test, open-field, light/dark box, and on a probabilistic reversal learning task. Results from the probabilistic reversal learning task are reported by Koloski et al. (2024). Data presented here are from the three-chamber social preference task, one of the most common methods to evaluate social behavior in rodents, which was run at 2, 6, and 10 weeks following TBI. Due to attrition of subjects, we are only able to evaluate data from the first time point. There was no habituation or training done in the social preference chamber prior to the initial testing day. However, due to the comprehensive behavioral battery, rats were habituated in an operant box and pretrained to nose poke for a water reward. Animals began testing 1 week after their electrophysiology implant surgery (2 weeks after the TBI). In rodents, 2 weeks post-TBI is past the acute phase of neuroinflammatory activation, hemorrhage, neural excitotoxicity, compromised blood brain barrier, and axonal shearing (<7 days) (Mao et al., 2020; Pearn et al., 2017; Risbrough et al., 2022) The progression of TBI is characterized by initial hemorrhages in the white matter beneath the contused cortex, which develops within the first hours postinjury (Feeney et al., 1981). The 2-week time point still captures a vulnerable state where functional changes in behavior may be influenced broadly by progressive brain atrophy, microglia activity, and diminished neural (Mao et al., 2020; Pearn et al., 2017; Risbrough et al., 2022). This intermediate time point allows us to assess functional outcomes during a critical window where both subacute recovery processes and early chronic changes are occurring, providing a balanced view of the effects of TBI on social behavior.

During the three-chamber sociability test, the subject was placed in the medial chamber and allowed to explore freely for 30 min. The lateral chambers contained a social (conspecific) and nonsocial stimulus (novel object: rubber duck). Social preference is the propensity for the subject to spend more time in the chamber with the animal compared with the novel object. A novel animal of the same age, strain, and sex was used as the social stimulus. Each test subject was presented with a new conspecific. The conspecific was habituated (10 min) in the containment 24 hr before the formal testing period. The subject was not habituated. The chamber and cage objects were cleaned with 70% ethanol between sessions.

### Histology

Twelve weeks postinjury, all rats were anesthetized with a lethal dose of isoflurane and sacrificed by transcardiac perfusion with 0.9% phosphate-buffered saline, followed by 4% phosphate-buffered formaldehyde. Brains were postfixed in 4% phosphate-buffered formaldehyde for 24 hr before being transferred to a 30% sucrose solution. Tissue was blocked in the flat skull position in 3-mm coronal sections, paraffin-embedded, and sectioned 20 μm thick on a microtome. Cut tissues were floated in a 40 °C hot water bath and mounted on slides. Slices were deparaffinized and stained with thionine to visualize cell body loss from injury and map electrode tracks. Sections were processed with a slide scanner at 40× magnification (Zeiss, Oberkochenn, Germany; Leica Biosystems, IL, USA). Figure 5C shows examples of Nissl-stained coronal sections from sham and TBI animals, the location of electrode tracks, and the maximum and minimum boundaries of injury. CCI-TBI resulted in prefrontal lesions at chronic time points, reduction in cell bodies, and increased gliosis. Although the impact was centered on the midline, the resulting chronic damage may not be perfectly centered. Notably, there was a 10-week gap between social preference recording and the perfusion/histology because the animals were subsequently trained and evaluated on additional cognitive tasks to complete a comprehensive series of behavioral assessments. Although this limits the conclusions, we can draw about brain pathology at the 2-week time point, collecting brain tissue samples immediately after the social task would have precluded these further assessments and limited our ability to gather a holistic view of TBI effects on cognitive functions over time (data for other tasks not mentioned here).

### Statistical Analysis

#### Behavioral analysis.

Behavioral data are publicly available (Open Data Commons for Traumatic Brain Injury database/NEATlabs—Ramanathan/Koloski). The location of animals was tracked using DeepLabCut (Mathis et al., 2018). We labeled six body parts on randomly selected frames (200) for each animal. Body parts labeled included are the snout, forepaws, hind paws, and tip of tail. After labeling, training on a deep neural network architecture was performed to allow for automated tracking of each object across frames. The output of this was a frame-by-frame X-Y coordinate map of each body part, along with an estimation of the confidence of the model in tracking that particular body part. After a review of these data, we decided to use the snout of the animal to identify its location within the maze. This decision was made based on a higher level of confidence in tracking compared with other body parts (~95% of detection points had a confidence index of 0.99 across animals) as well as the fact that where the animal’s head is in the maze is likely a more accurate representation of what it is exploring at any particular moment in time.

Social behaviors on this task were assessed by measuring the amount of time spent in each chamber, entries into each chamber, and the social preference index. Social preference was calculated using the following formula (Barzilay et al., 2011):

$$\text{Social}\;\text{Preference}=\frac{\text{Social}\;\text{Stimulus}\;\text{Interaction}\;\text{Time}-\text{NonSocial}\;\text{Stimulus}\;\text{Interaction}\;\text{Time}}{\text{Total}\;\text{exploration}\;\text{time}}$$

Locomotion was assessed by measuring traveling distance in all chambers throughout the entire session. Time spent (s) and the number of entries were analyzed with a mixed-effects ANOVA between the subject factor of the group (TBI vs. sham) and within the subject factor of the chamber (social vs. nonsocial). Significant effects (*p* < 0.05) were followed with post hoc *t*-test comparisons with Sidak’s correction for multiple comparisons. Social preference and distance traveled (cm) were analyzed as independent samples *t* tests (TBI vs. sham). The critical *p* value was set at 0.05, and data were represented as mean ± *SEM*. Statistical analyses were performed in SPSS, and visualizations were prepared using GraphPad Prism software.

#### Electrophysiology signals analysis.

LFP data are available (Koloski et al., 2024): thirty-two CH LFP recordings during social preference task in animals with bifrontal severe TBI (*DANDI Archive*, n.d.). LFP signals were processed offline using custom MATLAB scripts and functions from EEGLAB to ensure the removal of artifacts and enhance the quality of the data. Local referencing was performed using the means of the four adjacent electrodes on each shank. By utilizing the neighboring electrodes as a reference, common noise sources and spatial biases were minimized, allowing for a more accurate representation of the local LFP signals. This referencing approach accounted for the spatial distribution of the electrical potentials and helped to enhance the signal quality for further analysis and interpretation.

In the next step, the signal of each channel was normalized by the *z*-score method. For this purpose, the top 10% and bottom 10% of the signal samples were not used in calculating the average and standard deviation of the signal for each channel to minimize the effect of artifacts and outliers. Then, the power was calculated. Any channel that showed greater than 5 *SD* from the mean of all channels was discarded automatically. Next, we organized data into a set of “trials,” in which each trial represented a period in which an animal was in a particular chamber. Any trial with a signal >5 (reflecting a signal >5 *SD*) was discarded before further analysis to minimize the impacts of artifacts. This was followed by an analysis of power and coherence.

#### Power and coherence calculations.

To analyze power spectral density (PSD), the acquired signals were analyzed across the broad frequency range from 0.1 to 150 Hz using the pwelch MATLAB function with 1,000-ms sampling, 80% overlapping, and 0.5-Hz frequency resolution in all 32 channels. The mean power was then calculated across time for each electrode, within each chamber, and analyzed across groups/chambers. A series of independent *t* tests were carried out to examine differences in power between groups (sham vs. TBI) separately for each electrode at each frequency (0.1-Hz bins). Coherence was calculated within the gamma frequency band using the mscohere function in MATLAB, calculating the magnitude-squared coherence of two electrodes. To investigate coherence values between different chambers/groups, a 32 × 32 matrix was constructed with mean absolute values of the coherence between pairwise electrodes. Connectivity information was also graphically rendered as a circular diagram displaying relationships between pairs of regions while different values of coherence were encoded in the thickness of the connecting lines. For sorting the electrodes from high to low, we calculated RSS via the following formula:

$$RSS=\sqrt{\sum_{i+1}^{n}\sigma_{i}^{2}}$$

Statistical analyses were performed within SPSS or MATLAB (independent *t* test for PSD on a single channel and utilize ANOVA for analyzing PSD and coherence across multiple regions and chambers) and visualized using GraphPad Prism. Data are available on public repositories (DANDI and odc-TBI), and custom code is available on the author’s GitHub. Alpha of 0.05 is considered as significant, and Sidak’s multiple comparison correction was used for post hoc tests. To address the issue of multiple comparisons in our analysis, we used the FDR correction method.

## ACKNOWLEDGMENTS

We would like to thank Xuanyu Wu, Qiyu Chen, and Sidharth Hulyalkar for their contributions with electrophysiology data collection, processing, and analysis. This material is the result of work supported with resources and the use of facilities at the VA San Diego Medical Center and the Center of Excellence for Stress and Mental Health.

The contents of this manuscript do not represent the views of the U.S. Department of Veteran Affairs or the United States Government.

## SUPPORTING INFORMATION

Supporting information for this article is available at https://doi.org/10.1162/netn_a_00416.

## AUTHOR CONTRIBUTIONS

Morteza Salimi: Formal analysis; Methodology; Visualization; Writing – Original draft; Writing – review & editing. Tianzhi Tang: Data curation; Formal analysis; Methodology. Milad Nazari: Formal analysis; Methodology; Visualization; Writing – original draft. Jyoti Mishra: Conceptualization; Writing – review & editing. Houtan Afshar: Writing – original draft. Miranda Koloski: Conceptualization; Data curation; Formal analysis; Funding acquisition; Methodology; Project administration; Visualization; Writing – original draft; Writing – review & editing. Dhakshin Ramanathan: Conceptualization; Funding acquisition; Project administration; Supervision; Writing – original draft; Writing – review & editing.

## FUNDING INFORMATION

Dhakshin Ramanathan, Burroughs Wellcome Fund (https://dx.doi.org/10.13039/100000861), Award ID: 1015644. Dhakshin Ramanathan, National Institute of Mental Health (https://dx.doi.org/10.13039/100000025), Award ID: R01MH123650. Miranda Koloski, Foundation for the National Institutes of Health (https://dx.doi.org/10.13039/100000009), Award ID: T32-MH018300. Miranda Koloski, U.S. Department of Veterans Affairs (https://dx.doi.org/10.13039/100000738), Award ID: IK2BX006125.

## DATA AVAILABILITY STATEMENT

The data generated and analyzed during this study are available from the corresponding author upon reasonable request.

## Supplementary Material



## TECHNICAL TERMS

Traumatic brain injury: An injury to the head caused by an outside force that can cause damage immediately or gradually over the course of hours, days, or weeks following the injury.
Local field potential: Electrical activity generated by nerve cells that reflects the summed and synchronous activity of individual neurons in that area.
Controlled cortical impact: A model of brain injury that uses a controlled piston to induce a blow to the head whereby the size, depth, speed of impact, and dwell time are controlled by the experimenter.
Power spectrum: A representation of how much power or energy is contained within each frequency component.
Gamma frequency band: Gamma waves are the fastest neural oscillations that occur, with a frequency between 30 and 150 Hz.
Coherence: A method to determine if different areas of the brain have neuronal activity patterns that are correlated or not correlated.
Weighted connectivity: A network where the paths between nodes have weights assigned to them based on the degree of connectivity.
