# Supplementary material for: Gamma frequency connectivity in frontostriatal networks associated with social preference is reduced with traumatic brain injury
Source: Netw Neurosci. 2024 Dec 10;8(4):1634–53. doi: 10.1162/netn_a_00416 (PMC11675011; doi:10.1162/netn_a_00416)
Supplement: Supplementary file 1 [file netn-8-4-1634-s001.pdf]

**Supplementary figures.**

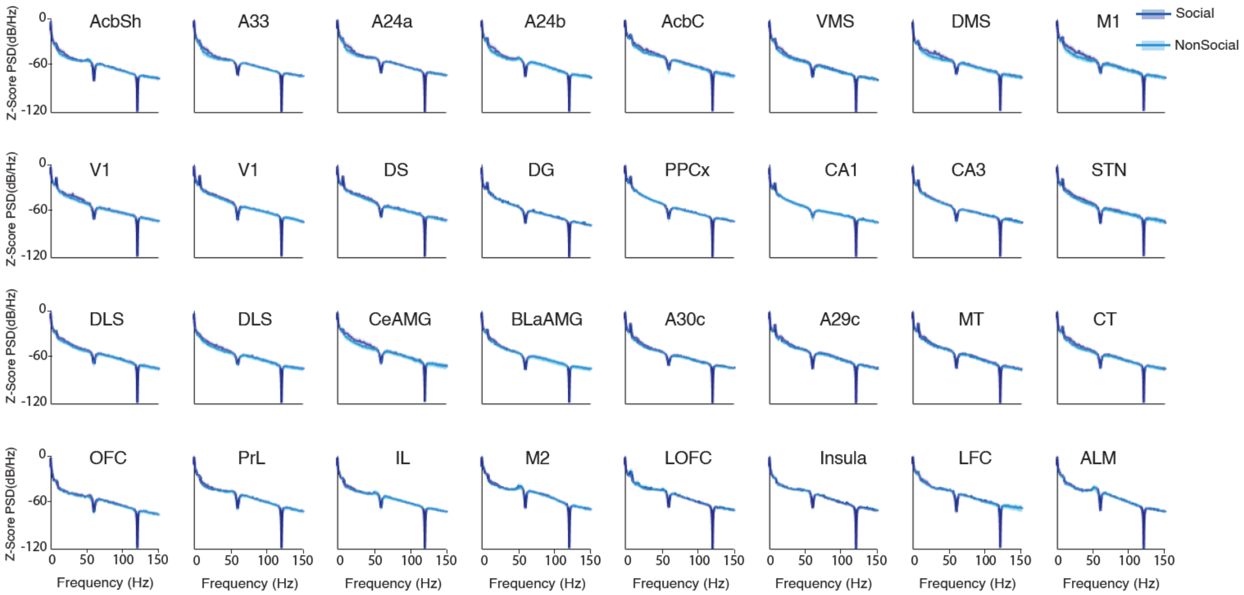


**Supplementary 1. Power Spectral Density in Sham animals.** Plots indicate the comparison between mean spectral power for Sham within social (dark blue) and non-social (light blue) in all 32 regions when exploring analyzed by paired-sample t-tests. Error bars represent SEM. There were no significant differences in power at any electrode location between social and non-social chambers. M2, motor area 2; Prl, prelimbic cortex; IL, infralimbic cortex; VO, ventral orbitofrontal cortex; M1, motor area 1; LFC, lateral frontal cortex; AI, Anterior Insula; LO, Lateral orbitofrontal cortex; DMS, dorsomedial striatum; VMS, ventromedial striatum; AcbC, nucleus accumbens core. AcbSh, nucleus accumbens shell; MT, medial thalamus; CT, central thalamus; DLS, dorsolateral subiculum; CeAMG, central amygdala; BLaAMG, basolateral amygdala; STN, subthalamic nucleus; CA3, Cornu Ammonis 3 hippocampus; CA1, Cornu Ammonis 1 hippocampus; PPx, Posterior parietal cortex; DG, dentate gyrus; V1, visual cortex 1.

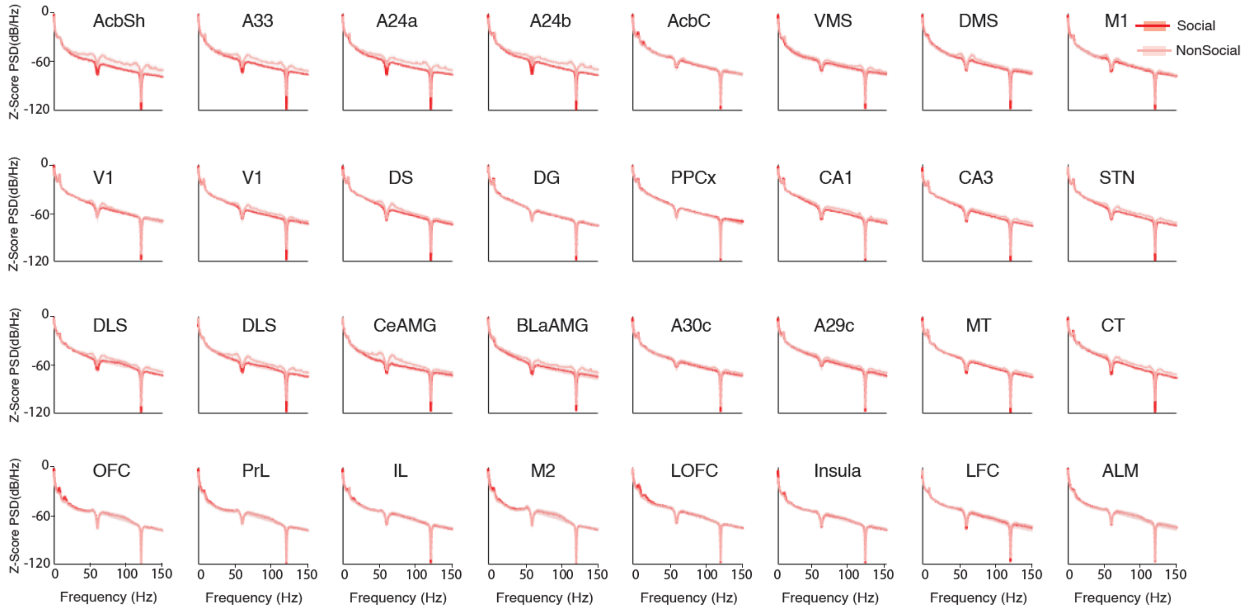


**Supplementary 2. Power Spectral Density in TBI animals.** Plots indicate the comparison between mean spectral power for TBI within social (dark red) and non-social (light red) in all 32 regions when exploring analyzed by paired-sample t-tests. Error bars represent SEM. There were no significant differences at any electrode location between power in social and non-social chambers. M2, motor area 2; Prl, prelimbic cortex; IL, infralimbic cortex; VO, ventral orbitofrontal cortex; M1, motor area 1; LFC, lateral frontal cortex; AI, Anterior Insula; LO, Lateral orbitofrontal cortex; VMS, ventromedial striatum; DMS, dorsomedial striatum; AcbC, nucleus accumbens core; AcbSh, nucleus accumbens shell; MT, medial thalamus; CT, central thalamus; DLS, dorsolateral subiculum; CeAMG, central amygdala; BLaAMG, basolateral amygdala; STN, subthalamic nucleus; CA3, Cornu Ammonis 3 hippocampus; CA1, Cornu Ammonis 1 hippocampus; PPx, Posterior parietal cortex; DG, dentate gyrus; V1, visual cortex 1.

| | AcbSh | A33 | A24a | A24b | AcbC | VMS | DMS | M1 | V1 | V1 | DS | DG | PPx | CA1 | CA3 | STN | DLS | DLS | CeAMG | BlaAMG | A30c | A29c | MT | CT | OFC | IL | PrL | M2 | LOFC | Insula | LFC | ALM |
|---|---|---|---|---|---|---|---|---|---|---|---|---|---|---|---|---|---|---|---|---|---|---|---|---|---|---|---|---|---|---|---|---|
| AcbSh | | 0.155 | 0.391 | 0.337 | 0.185 | 0.279 | 0.061 | 0.029 | 0.018 | 0.010 | 0.019 | 0.011 | 0.015 | 0.010 | 0.033 | 0.016 | 0.129 | 0.015 | 0.043 | 0.042 | 0.342 | 0.383 | 0.279 | 0.158 | 0.063 | 0.094 | 0.259 | 0.168 | 0.139 | 0.168 | 0.295 | 0.268 |
| A33 | 0.155 | | 0.152 | 0.197 | 0.061 | 0.307 | 0.009 | 0.010 | 0.012 | 0.055 | 0.014 | 0.056 | 0.010 | 0.010 | 0.010 | 0.009 | 0.013 | 0.017 | 0.010 | 0.181 | 0.085 | 0.046 | 0.073 | 0.206 | 0.009 | 0.010 | 0.316 | 0.064 | 0.012 | 0.011 | 0.206 | 0.208 |
| A24a | 0.391 | 0.152 | | 0.404 | 0.038 | 0.345 | 0.016 | 0.069 | 0.010 | 0.010 | 0.010 | 0.010 | 0.069 | 0.010 | 0.029 | 0.029 | 0.212 | 0.073 | 0.020 | 0.273 | 0.304 | 0.206 | 0.273 | 0.193 | 0.018 | 0.038 | 0.357 | 0.129 | 0.032 | 0.027 | 0.277 | 0.131 |
| A24b | 0.337 | 0.197 | 0.404 | | 0.028 | 0.145 | 0.010 | 0.113 | 0.015 | 0.010 | 0.013 | 0.010 | 0.009 | 0.010 | 0.023 | 0.010 | 0.019 | 0.009 | 0.011 | 0.166 | 0.314 | 0.223 | 0.316 | 0.238 | 0.008 | 0.046 | 0.161 | 0.086 | 0.034 | 0.024 | 0.380 | 0.042 |
| AcbC | 0.185 | 0.061 | 0.038 | 0.028 | | 0.038 | 0.041 | 0.097 | 0.023 | 0.009 | 0.024 | 0.009 | 0.009 | 0.011 | 0.010 | 0.018 | 0.008 | 0.008 | 0.009 | 0.015 | 0.023 | 0.010 | 0.019 | 0.130 | 0.009 | 0.009 | 0.027 | 0.008 | 0.009 | 0.008 | 0.020 | 0.020 |
| VMS | 0.279 | 0.307 | 0.345 | 0.145 | 0.038 | | 0.043 | 0.212 | 0.010 | 0.010 | 0.010 | 0.011 | 0.009 | 0.010 | 0.086 | 0.014 | 0.010 | 0.011 | 0.009 | 0.249 | 0.256 | 0.241 | 0.382 | 0.350 | 0.014 | 0.165 | 0.218 | 0.081 | 0.015 | 0.008 | 0.472 | 0.279 |
| DMS | 0.061 | 0.009 | 0.016 | 0.010 | 0.041 | 0.043 | | 0.135 | 0.168 | 0.009 | 0.078 | 0.009 | 0.010 | 0.009 | 0.010 | 0.066 | 0.009 | 0.221 | 0.034 | 0.014 | 0.023 | 0.030 | 0.025 | 0.085 | 0.038 | 0.026 | 0.009 | 0.007 | 0.019 | 0.038 | 0.021 | 0.010 |
| M1 | 0.029 | 0.010 | 0.069 | 0.113 | 0.097 | 0.212 | 0.135 | | 0.029 | 0.009 | 0.020 | 0.009 | 0.020 | 0.011 | 0.025 | 0.059 | 0.009 | 0.015 | 0.038 | 0.021 | 0.026 | 0.028 | 0.073 | 0.170 | 0.044 | 0.256 | 0.030 | 0.010 | 0.009 | 0.012 | 0.073 | 0.106 |
| V1 | 0.018 | 0.012 | 0.010 | 0.015 | 0.023 | 0.010 | 0.168 | 0.029 | | 0.387 | 0.507 | 0.373 | 0.014 | 0.018 | 0.011 | 0.009 | 0.014 | 0.122 | 0.223 | 0.015 | 0.009 | 0.012 | 0.009 | 0.011 | 0.009 | 0.009 | 0.009 | 0.009 | 0.013 | 0.054 | 0.009 | 0.009 |
| V1 | 0.010 | 0.055 | 0.010 | 0.010 | 0.009 | 0.010 | 0.009 | 0.009 | 0.387 | | 0.345 | 0.448 | 0.015 | 0.142 | 0.023 | 0.064 | 0.019 | 0.019 | 0.015 | 0.028 | 0.030 | 0.038 | 0.029 | 0.026 | 0.014 | 0.009 | 0.010 | 0.009 | 0.009 | 0.015 | 0.010 | 0.011 |
| DS | 0.019 | 0.014 | 0.010 | 0.013 | 0.024 | 0.010 | 0.078 | 0.020 | 0.507 | 0.345 | | 0.273 | 0.043 | 0.075 | 0.043 | 0.060 | 0.024 | 0.048 | 0.239 | 0.013 | 0.018 | 0.043 | 0.015 | 0.022 | 0.009 | 0.009 | 0.010 | 0.009 | 0.054 | 0.097 | 0.010 | 0.010 |
| DG | 0.011 | 0.056 | 0.010 | 0.010 | 0.009 | 0.011 | 0.009 | 0.009 | 0.373 | 0.448 | 0.273 | | 0.009 | 0.020 | 0.010 | 0.018 | 0.010 | 0.012 | 0.010 | 0.011 | 0.021 | 0.020 | 0.010 | 0.022 | 0.009 | 0.009 | 0.009 | 0.009 | 0.010 | 0.020 | 0.010 | 0.010 |
| PPx | 0.015 | 0.010 | 0.069 | 0.009 | 0.009 | 0.009 | 0.010 | 0.020 | 0.014 | 0.015 | 0.043 | 0.009 | | 0.460 | 0.265 | 0.375 | 0.057 | 0.025 | 0.011 | 0.022 | 0.020 | 0.013 | 0.020 | 0.015 | 0.010 | 0.009 | 0.009 | 0.008 | 0.009 | 0.009 | 0.009 | 0.008 |
| CA1 | 0.010 | 0.010 | 0.010 | 0.010 | 0.011 | 0.010 | 0.009 | 0.011 | 0.018 | 0.142 | 0.075 | 0.020 | 0.460 | | 0.279 | 0.309 | 0.073 | 0.080 | 0.046 | 0.160 | 0.132 | 0.155 | 0.166 | 0.102 | 0.010 | 0.010 | 0.010 | 0.010 | 0.009 | 0.009 | 0.010 | 0.010 |
| CA3 | 0.033 | 0.010 | 0.029 | 0.023 | 0.010 | 0.086 | 0.010 | 0.025 | 0.011 | 0.023 | 0.043 | 0.010 | 0.265 | 0.279 | | 0.351 | 0.019 | 0.015 | 0.009 | 0.177 | 0.083 | 0.057 | 0.104 | 0.049 | 0.049 | 0.069 | 0.035 | 0.030 | 0.009 | 0.009 | 0.022 | 0.023 |
| STN | 0.016 | 0.009 | 0.029 | 0.010 | 0.018 | 0.014 | 0.066 | 0.059 | 0.009 | 0.064 | 0.060 | 0.018 | 0.375 | 0.309 | 0.351 | | 0.020 | 0.028 | 0.024 | 0.061 | 0.075 | 0.064 | 0.094 | 0.056 | 0.177 | 0.083 | 0.010 | 0.011 | 0.009 | 0.009 | 0.015 | 0.009 |
| DLS | 0.129 | 0.013 | 0.212 | 0.019 | 0.008 | 0.010 | 0.009 | 0.009 | 0.014 | 0.019 | 0.024 | 0.010 | 0.057 | 0.073 | 0.019 | 0.020 | | 0.279 | 0.354 | 0.326 | 0.114 | 0.208 | 0.098 | 0.086 | 0.008 | 0.009 | 0.009 | 0.032 | 0.046 | 0.075 | 0.064 | 0.047 |
| DLS | 0.015 | 0.017 | 0.073 | 0.009 | 0.008 | 0.011 | 0.221 | 0.015 | 0.122 | 0.019 | 0.048 | 0.012 | 0.025 | 0.080 | 0.015 | 0.028 | 0.279 | | 0.449 | 0.319 | 0.086 | 0.218 | 0.101 | 0.094 | 0.009 | 0.009 | 0.008 | 0.008 | 0.009 | 0.010 | 0.009 | 0.008 |
| CeAMG | 0.043 | 0.010 | 0.020 | 0.011 | 0.009 | 0.009 | 0.034 | 0.038 | 0.223 | 0.015 | 0.239 | 0.010 | 0.011 | 0.046 | 0.009 | 0.024 | 0.354 | 0.449 | | 0.353 | 0.087 | 0.200 | 0.061 | 0.094 | 0.009 | 0.010 | 0.010 | 0.075 | 0.152 | 0.075 | 0.014 | 0.034 |
| BlaAMG | 0.042 | 0.181 | 0.273 | 0.166 | 0.015 | 0.249 | 0.014 | 0.021 | 0.015 | 0.028 | 0.013 | 0.011 | 0.022 | 0.160 | 0.177 | 0.061 | 0.326 | 0.319 | 0.353 | | 0.301 | 0.403 | 0.346 | 0.277 | 0.009 | 0.087 | 0.251 | 0.043 | 0.009 | 0.009 | 0.404 | 0.218 |
| A30c | 0.342 | 0.085 | 0.304 | 0.314 | 0.023 | 0.256 | 0.023 | 0.026 | 0.009 | 0.030 | 0.018 | 0.021 | 0.020 | 0.132 | 0.083 | 0.075 | 0.114 | 0.086 | 0.087 | 0.301 | | 0.652 | 0.461 | 0.311 | 0.009 | 0.123 | 0.361 | 0.074 | 0.015 | 0.009 | 0.208 | 0.081 |
| A29c | 0.383 | 0.046 | 0.206 | 0.223 | 0.010 | 0.241 | 0.030 | 0.028 | 0.012 | 0.038 | 0.043 | 0.020 | 0.013 | 0.155 | 0.057 | 0.064 | 0.208 | 0.218 | 0.200 | 0.403 | 0.652 | | 0.445 | 0.250 | 0.009 | 0.036 | 0.248 | 0.042 | 0.023 | 0.046 | 0.104 | 0.018 |
| MT | 0.279 | 0.073 | 0.273 | 0.316 | 0.019 | 0.382 | 0.025 | 0.073 | 0.009 | 0.029 | 0.015 | 0.010 | 0.020 | 0.166 | 0.104 | 0.094 | 0.098 | 0.101 | 0.061 | 0.346 | 0.461 | 0.445 | | 0.279 | 0.011 | 0.160 | 0.366 | 0.108 | 0.011 | 0.009 | 0.274 | 0.130 |
| CT | 0.158 | 0.206 | 0.193 | 0.238 | 0.130 | 0.350 | 0.085 | 0.170 | 0.011 | 0.026 | 0.022 | 0.022 | 0.015 | 0.102 | 0.049 | 0.056 | 0.086 | 0.094 | 0.094 | 0.277 | 0.311 | 0.250 | 0.279 | | 0.012 | 0.252 | 0.460 | 0.146 | 0.009 | 0.010 | 0.273 | 0.326 |
| OFC | 0.063 | 0.009 | 0.018 | 0.008 | 0.009 | 0.014 | 0.038 | 0.044 | 0.009 | 0.014 | 0.009 | 0.009 | 0.010 | 0.010 | 0.049 | 0.177 | 0.008 | 0.009 | 0.009 | 0.009 | 0.009 | 0.009 | 0.011 | 0.012 | | 0.230 | 0.139 | 0.192 | 0.019 | 0.021 | 0.065 | 0.170 |
| IL | 0.094 | 0.010 | 0.038 | 0.046 | 0.009 | 0.165 | 0.026 | 0.256 | 0.009 | 0.009 | 0.009 | 0.009 | 0.009 | 0.010 | 0.069 | 0.083 | 0.009 | 0.009 | 0.010 | 0.087 | 0.123 | 0.036 | 0.160 | 0.252 | 0.230 | | 0.545 | 0.090 | 0.043 | 0.011 | 0.160 | 0.218 |
| PrL | 0.259 | 0.316 | 0.357 | 0.161 | 0.027 | 0.218 | 0.009 | 0.030 | 0.009 | 0.010 | 0.010 | 0.009 | 0.009 | 0.010 | 0.035 | 0.010 | 0.009 | 0.008 | 0.010 | 0.251 | 0.361 | 0.248 | 0.366 | 0.460 | 0.139 | 0.545 | | 0.239 | 0.019 | 0.043 | 0.345 | 0.316 |
| M2 | 0.168 | 0.064 | 0.129 | 0.086 | 0.008 | 0.081 | 0.007 | 0.010 | 0.009 | 0.009 | 0.009 | 0.009 | 0.008 | 0.010 | 0.030 | 0.011 | 0.032 | 0.008 | 0.075 | 0.043 | 0.074 | 0.042 | 0.108 | 0.146 | 0.192 | 0.090 | 0.239 | | 0.175 | 0.130 | 0.160 | 0.333 |
| LOFC | 0.139 | 0.012 | 0.032 | 0.034 | 0.009 | 0.015 | 0.019 | 0.009 | 0.013 | 0.009 | 0.054 | 0.010 | 0.009 | 0.009 | 0.009 | 0.009 | 0.046 | 0.009 | 0.152 | 0.009 | 0.015 | 0.023 | 0.011 | 0.009 | 0.019 | 0.043 | 0.019 | 0.175 | | 0.386 | 0.315 | 0.273 |
| Insula | 0.168 | 0.011 | 0.027 | 0.024 | 0.008 | 0.008 | 0.038 | 0.012 | 0.054 | 0.015 | 0.097 | 0.020 | 0.009 | 0.009 | 0.009 | 0.009 | 0.075 | 0.010 | 0.075 | 0.009 | 0.009 | 0.046 | 0.009 | 0.010 | 0.021 | 0.011 | 0.043 | 0.130 | 0.386 | | 0.388 | 0.294 |
| LFC | 0.295 | 0.206 | 0.277 | 0.380 | 0.020 | 0.472 | 0.021 | 0.073 | 0.009 | 0.010 | 0.010 | 0.010 | 0.009 | 0.010 | 0.022 | 0.015 | 0.064 | 0.009 | 0.014 | 0.404 | 0.208 | 0.104 | 0.274 | 0.273 | 0.065 | 0.160 | 0.345 | 0.160 | 0.315 | 0.388 | | 0.392 |
| ALM | 0.268 | 0.208 | 0.131 | 0.042 | 0.020 | 0.279 | 0.010 | 0.106 | 0.009 | 0.011 | 0.010 | 0.010 | 0.008 | 0.010 | 0.023 | 0.009 | 0.047 | 0.008 | 0.034 | 0.218 | 0.081 | 0.018 | 0.130 | 0.326 | 0.170 | 0.218 | 0.316 | 0.333 | 0.273 | 0.294 | 0.392 | |

**Supplementary 3. Quantity of p-values for analyzing the coherence between sham animals during periods of social and nonsocial preference.** M2, motor area 2; Prl, prelimbic cortex; IL, infralimbic cortex; VO, ventral orbitofrontal cortex; M1, motor area 1; LFC, lateral frontal cortex; AI, Anterior Insula; LO, Lateral orbitofrontal cortex; DMS, dorsomedial striatum; VMS, ventromedial striatum; AcbC, nucleus accumbens core; AcbSh, nucleus accumbens shell; MT, medial thalamus; CT, central thalamus; DLS, dorsolateral subiculum; CeAMG, central amygdala; BLaAMG, basolateral amygdala; STN, subthalamic nucleus; CA3, Cornu Ammonis 3 hippocampus; CA1, Cornu Ammonis 1 hippocampus; PPx, Posterior parietal cortex; DG, dentate gyrus; V1, visual cortex 1.

| | AcbSh | A33 | A24a | A24b | AcbC | VMS | DMS | M1 | V1 | V1 | DS | DG | PPx | CA1 | CA3 | STN | DLS | DLS | CeAMG | BlaAMG | A30c | A29c | MT | CT | OFC | IL | PrL | M2 | LOFC | Insula | LFC | ALM |
|---|---|---|---|---|---|---|---|---|---|---|---|---|---|---|---|---|---|---|---|---|---|---|---|---|---|---|---|---|---|---|---|---|
| AcbSh | | 0.479 | 0.869 | 0.968 | 0.629 | 0.940 | 0.390 | 0.571 | 0.583 | 0.500 | 0.577 | 0.525 | 0.540 | 0.358 | 0.364 | 0.451 | 0.924 | 0.762 | 0.793 | 0.649 | 0.682 | 0.955 | 0.643 | 0.574 | 0.604 | 0.708 | 0.842 | 0.590 | 0.650 | 0.882 | 0.682 | 0.948 |
| A33 | 0.479 | | 0.681 | 0.666 | 0.973 | 0.903 | 0.413 | 0.640 | 0.519 | 0.604 | 0.398 | 0.612 | 0.489 | 0.359 | 0.369 | 0.318 | 0.553 | 0.660 | 0.464 | 0.531 | 0.652 | 0.498 | 0.621 | 0.993 | 0.291 | 0.582 | 0.956 | 0.611 | 0.519 | 0.463 | 0.735 | 0.831 |
| A24a | 0.869 | 0.681 | | 0.984 | 0.730 | 0.759 | 0.533 | 0.694 | 0.579 | 0.560 | 0.544 | 0.525 | 0.459 | 0.698 | 0.398 | 0.398 | 0.705 | 0.489 | 0.823 | 0.632 | 0.995 | 0.861 | 0.911 | 0.726 | 0.799 | 0.474 | 0.790 | 0.545 | 0.451 | 0.503 | 0.624 | 0.514 |
| A24b | 0.968 | 0.666 | 0.984 | | 0.757 | 0.740 | 0.566 | 0.604 | 0.350 | 0.403 | 0.483 | 0.449 | 0.596 | 0.324 | 0.633 | 0.377 | 0.774 | 0.502 | 0.497 | 0.925 | 0.893 | 0.875 | 0.815 | 0.763 | 0.445 | 0.556 | 0.901 | 0.838 | 0.573 | 0.731 | 0.828 | 0.696 |
| AcbC | 0.629 | 0.973 | 0.730 | 0.757 | | 0.561 | 0.839 | 0.968 | 0.822 | 0.590 | 0.677 | 0.475 | 0.569 | 0.410 | 0.316 | 0.588 | 0.606 | 0.735 | 0.732 | 0.471 | 0.544 | 0.421 | 0.505 | 0.980 | 0.752 | 0.615 | 0.859 | 0.824 | 0.553 | 0.435 | 0.738 | 0.911 |
| VMS | 0.940 | 0.903 | 0.759 | 0.740 | 0.561 | | 0.295 | 0.725 | 0.491 | 0.541 | 0.463 | 0.588 | 0.414 | 0.321 | 0.363 | 0.415 | 0.388 | 0.440 | 0.556 | 0.684 | 0.548 | 0.507 | 0.712 | 0.820 | 0.494 | 0.578 | 0.955 | 0.583 | 0.468 | 0.463 | 0.931 | 0.813 |
| DMS | 0.390 | 0.413 | 0.533 | 0.566 | 0.839 | 0.295 | | 0.826 | 0.984 | 0.646 | 0.942 | 0.757 | 0.684 | 0.510 | 0.402 | 0.689 | 0.586 | 0.940 | 0.774 | 0.681 | 0.423 | 0.499 | 0.677 | 0.724 | 0.694 | 0.612 | 0.487 | 0.665 | 0.508 | 0.649 | 0.518 | 0.820 |
| M1 | 0.571 | 0.640 | 0.694 | 0.604 | 0.968 | 0.725 | 0.826 | | 0.801 | 0.609 | 0.802 | 0.472 | 0.695 | 0.560 | 0.386 | 0.655 | 0.536 | 0.787 | 0.870 | 0.459 | 0.484 | 0.493 | 0.449 | 0.874 | 0.844 | 0.854 | 0.602 | 0.759 | 0.589 | 0.603 | 0.691 | 0.954 |
| V1 | 0.583 | 0.519 | 0.579 | 0.350 | 0.822 | 0.491 | 0.984 | 0.801 | | 0.720 | 0.938 | 0.709 | 0.753 | 0.592 | 0.382 | 0.719 | 0.599 | 0.886 | 0.946 | 0.512 | 0.450 | 0.600 | 0.605 | 0.684 | 0.682 | 0.595 | 0.416 | 0.409 | 0.510 | 0.720 | 0.386 | 0.626 |
| V1 | 0.500 | 0.604 | 0.560 | 0.403 | 0.590 | 0.541 | 0.646 | 0.609 | 0.720 | | 0.653 | 0.976 | 0.557 | 0.736 | 0.511 | 0.621 | 0.499 | 0.483 | 0.664 | 0.420 | 0.379 | 0.576 | 0.541 | 0.553 | 0.415 | 0.383 | 0.381 | 0.400 | 0.423 | 0.509 | 0.378 | 0.642 |
| DS | 0.577 | 0.398 | 0.544 | 0.483 | 0.677 | 0.463 | 0.942 | 0.802 | 0.938 | 0.653 | | 0.943 | 0.800 | 0.577 | 0.524 | 0.782 | 0.629 | 0.767 | 0.915 | 0.536 | 0.320 | 0.632 | 0.637 | 0.538 | 0.585 | 0.658 | 0.352 | 0.462 | 0.639 | 0.779 | 0.471 | 0.719 |
| DG | 0.525 | 0.612 | 0.525 | 0.449 | 0.475 | 0.588 | 0.757 | 0.472 | 0.709 | 0.976 | 0.943 | | 0.403 | 0.558 | 0.454 | 0.567 | 0.565 | 0.444 | 0.717 | 0.422 | 0.394 | 0.540 | 0.659 | 0.410 | 0.536 | 0.373 | 0.369 | 0.387 | 0.480 | 0.623 | 0.410 | 0.632 |
| PPx | 0.540 | 0.489 | 0.459 | 0.596 | 0.569 | 0.414 | 0.684 | 0.695 | 0.753 | 0.557 | 0.800 | 0.403 | | 0.618 | 0.512 | 0.784 | 0.487 | 0.845 | 0.586 | 0.497 | 0.488 | 0.579 | 0.373 | 0.572 | 0.700 | 0.640 | 0.386 | 0.502 | 0.435 | 0.580 | 0.433 | 0.565 |
| CA1 | 0.358 | 0.359 | 0.698 | 0.324 | 0.410 | 0.321 | 0.510 | 0.560 | 0.592 | 0.736 | 0.577 | 0.558 | 0.618 | | 0.803 | 0.381 | 0.455 | 0.461 | 0.581 | 0.394 | 0.394 | 0.437 | 0.530 | 0.593 | 0.478 | 0.357 | 0.356 | 0.316 | 0.323 | 0.299 | 0.359 | 0.290 |
| CA3 | 0.364 | 0.369 | 0.398 | 0.633 | 0.316 | 0.363 | 0.402 | 0.386 | 0.382 | 0.511 | 0.524 | 0.454 | 0.512 | 0.803 | | 0.544 | 0.312 | 0.324 | 0.320 | 0.574 | 0.503 | 0.564 | 0.355 | 0.450 | 0.365 | 0.408 | 0.327 | 0.457 | 0.305 | 0.336 | 0.348 | 0.354 |
| STN | 0.451 | 0.318 | 0.398 | 0.377 | 0.588 | 0.415 | 0.689 | 0.655 | 0.719 | 0.621 | 0.782 | 0.567 | 0.784 | 0.381 | 0.544 | | 0.294 | 0.610 | 0.529 | 0.332 | 0.456 | 0.458 | 0.412 | 0.555 | 0.705 | 0.645 | 0.398 | 0.476 | 0.364 | 0.344 | 0.381 | 0.408 |
| DLS | 0.924 | 0.553 | 0.705 | 0.774 | 0.606 | 0.388 | 0.586 | 0.536 | 0.599 | 0.499 | 0.629 | 0.565 | 0.487 | 0.455 | 0.312 | 0.294 | | 0.895 | 0.672 | 0.711 | 0.474 | 0.895 | 0.598 | 0.490 | 0.743 | 0.834 | 0.862 | 0.719 | 0.813 | 0.894 | 0.750 | 0.755 |
| DLS | 0.762 | 0.660 | 0.489 | 0.502 | 0.735 | 0.440 | 0.940 | 0.787 | 0.886 | 0.483 | 0.767 | 0.444 | 0.845 | 0.461 | 0.324 | 0.610 | 0.895 | | 0.659 | 0.776 | 0.412 | 0.614 | 0.369 | 0.564 | 0.676 | 0.829 | 0.723 | 0.570 | 0.618 | 0.714 | 0.630 | 0.576 |
| CeAMG | 0.793 | 0.464 | 0.823 | 0.497 | 0.732 | 0.556 | 0.774 | 0.870 | 0.946 | 0.664 | 0.915 | 0.717 | 0.586 | 0.581 | 0.320 | 0.529 | 0.672 | 0.659 | | 0.657 | 0.426 | 0.818 | 0.678 | 0.407 | 0.877 | 0.734 | 0.478 | 0.430 | 0.752 | 0.768 | 0.495 | 0.616 |
| BlaAMG | 0.649 | 0.531 | 0.632 | 0.925 | 0.471 | 0.684 | 0.681 | 0.459 | 0.512 | 0.420 | 0.536 | 0.422 | 0.497 | 0.394 | 0.574 | 0.332 | 0.711 | 0.776 | 0.657 | | 0.386 | 0.780 | 0.514 | 0.551 | 0.417 | 0.542 | 0.982 | 0.676 | 0.500 | 0.640 | 0.742 | 0.726 |
| A30c | 0.682 | 0.652 | 0.995 | 0.893 | 0.544 | 0.548 | 0.423 | 0.484 | 0.450 | 0.379 | 0.320 | 0.394 | 0.488 | 0.394 | 0.503 | 0.456 | 0.474 | 0.412 | 0.426 | 0.386 | | 0.981 | 0.932 | 0.657 | 0.365 | 0.402 | 0.901 | 0.508 | 0.330 | 0.379 | 0.452 | 0.381 |
| A29c | 0.955 | 0.498 | 0.861 | 0.875 | 0.421 | 0.507 | 0.499 | 0.493 | 0.600 | 0.576 | 0.632 | 0.540 | 0.579 | 0.437 | 0.564 | 0.458 | 0.895 | 0.614 | 0.818 | 0.780 | 0.981 | | 0.847 | 0.368 | 0.536 | 0.446 | 0.689 | 0.485 | 0.510 | 0.807 | 0.495 | 0.411 |
| MT | 0.643 | 0.621 | 0.911 | 0.815 | 0.505 | 0.712 | 0.677 | 0.449 | 0.605 | 0.541 | 0.637 | 0.659 | 0.373 | 0.530 | 0.355 | 0.412 | 0.598 | 0.369 | 0.678 | 0.514 | 0.932 | 0.847 | | 0.796 | 0.579 | 0.400 | 0.857 | 0.542 | 0.311 | 0.369 | 0.506 | 0.391 |
| CT | 0.574 | 0.993 | 0.726 | 0.763 | 0.980 | 0.820 | 0.724 | 0.874 | 0.684 | 0.553 | 0.538 | 0.410 | 0.572 | 0.593 | 0.450 | 0.555 | 0.490 | 0.564 | 0.407 | 0.551 | 0.657 | 0.368 | 0.796 | | 0.394 | 0.595 | 0.951 | 0.708 | 0.416 | 0.429 | 0.702 | 0.718 |
| OFC | 0.604 | 0.291 | 0.799 | 0.445 | 0.752 | 0.494 | 0.694 | 0.844 | 0.682 | 0.415 | 0.585 | 0.536 | 0.700 | 0.478 | 0.365 | 0.705 | 0.743 | 0.676 | 0.877 | 0.417 | 0.365 | 0.536 | 0.579 | 0.394 | | 0.848 | 0.580 | 0.746 | 0.685 | 0.728 | 0.539 | 0.881 |
| IL | 0.708 | 0.582 | 0.474 | 0.556 | 0.615 | 0.578 | 0.612 | 0.854 | 0.595 | 0.383 | 0.658 | 0.373 | 0.640 | 0.357 | 0.408 | 0.645 | 0.834 | 0.829 | 0.734 | 0.542 | 0.402 | 0.446 | 0.400 | 0.595 | 0.848 | | 0.824 | 0.576 | 0.793 | 0.800 | 0.870 | 0.879 |
| PrL | 0.842 | 0.956 | 0.790 | 0.901 | 0.859 | 0.955 | 0.487 | 0.602 | 0.416 | 0.381 | 0.352 | 0.369 | 0.386 | 0.356 | 0.327 | 0.398 | 0.862 | 0.723 | 0.478 | 0.982 | 0.901 | 0.689 | 0.857 | 0.951 | 0.580 | 0.824 | | 0.627 | 0.591 | 0.628 | 0.812 | 0.849 |
| M2 | 0.590 | 0.611 | 0.545 | 0.838 | 0.824 | 0.583 | 0.665 | 0.759 | 0.409 | 0.400 | 0.462 | 0.387 | 0.502 | 0.316 | 0.457 | 0.476 | 0.719 | 0.570 | 0.430 | 0.676 | 0.508 | 0.485 | 0.542 | 0.708 | 0.746 | 0.576 | 0.627 | | 0.680 | 0.744 | 0.732 | 0.955 |
| LOFC | 0.650 | 0.519 | 0.451 | 0.573 | 0.553 | 0.468 | 0.508 | 0.589 | 0.510 | 0.423 | 0.639 | 0.480 | 0.435 | 0.323 | 0.305 | 0.364 | 0.813 | 0.618 | 0.752 | 0.500 | 0.330 | 0.510 | 0.311 | 0.416 | 0.685 | 0.793 | 0.591 | 0.680 | | 0.926 | 0.954 | 0.845 |
| Insula | 0.882 | 0.463 | 0.503 | 0.731 | 0.435 | 0.463 | 0.649 | 0.603 | 0.720 | 0.509 | 0.779 | 0.623 | 0.580 | 0.299 | 0.336 | 0.344 | 0.894 | 0.714 | 0.768 | 0.640 | 0.379 | 0.807 | 0.369 | 0.429 | 0.728 | 0.800 | 0.628 | 0.744 | 0.926 | | 0.809 | 0.973 |
| LFC | 0.682 | 0.735 | 0.624 | 0.828 | 0.738 | 0.931 | 0.518 | 0.691 | 0.386 | 0.378 | 0.471 | 0.410 | 0.433 | 0.359 | 0.348 | 0.381 | 0.750 | 0.630 | 0.495 | 0.742 | 0.452 | 0.495 | 0.506 | 0.702 | 0.539 | 0.870 | 0.812 | 0.732 | 0.954 | 0.809 | | 0.868 |
| ALM | 0.948 | 0.831 | 0.514 | 0.696 | 0.911 | 0.813 | 0.820 | 0.954 | 0.626 | 0.642 | 0.719 | 0.632 | 0.565 | 0.290 | 0.354 | 0.408 | 0.755 | 0.576 | 0.616 | 0.726 | 0.381 | 0.411 | 0.391 | 0.718 | 0.881 | 0.879 | 0.849 | 0.955 | 0.845 | 0.973 | 0.868 | |

**Supplementary 4. Quantity of p-values for analyzing the coherence between TBI animals during periods of social and nonsocial preference.** M2, motor area 2; Prl, prelimbic cortex; IL, infralimbic cortex; VO, ventral orbitofrontal cortex; M1, motor area 1; LFC, lateral frontal cortex; AI, Anterior Insula; LO, Lateral orbitofrontal cortex; DMS, dorsomedial striatum; VMS, ventromedial striatum; AcbC, nucleus accumbens core; AcbSh, nucleus accumbens shell; MT, medial thalamus; CT, central thalamus; DLS, dorsolateral subiculum; CeAMG, central amygdala; BLaAMG, basolateral amygdala; STN, subthalamic nucleus; CA3, Cornu Ammonis 3 hippocampus; CA1, Cornu Ammonis 1 hippocampus; PPx, Posterior parietal cortex; DG, dentate gyrus; V1, visual cortex 1.

| | AcbSh | A33 | A24a | A24b | AcbC | VMS | DMS | M1 | V1 | V1 | DS | DG | PPx | CA1 | CA3 | STN |
|---|---|---|---|---|---|---|---|---|---|---|---|---|---|---|---|---|
| AcbSh | | 0.031 | 0.024 | 0.093 | 0.203 | 0.002 | 0.001 | 0.020 | 0.056 | 0.068 | 0.059 | 0.097 | 0.046 | 0.154 | 0.041 | 0.085 |
| A33 | 0.031 | | 0.086 | 0.278 | 0.119 | 0.303 | 0.065 | 0.055 | 0.073 | 0.140 | 0.086 | 0.105 | 0.034 | 0.223 | 0.045 | 0.028 |
| A24a | 0.024 | 0.086 | | 0.356 | 0.264 | 0.516 | 0.317 | 0.604 | 0.454 | 0.563 | 0.388 | 0.330 | 0.061 | 0.931 | 0.162 | 0.140 |
| A24b | 0.024 | 0.278 | 0.356 | | 0.042 | 0.003 | 0.042 | 0.022 | 0.333 | 0.269 | 0.355 | 0.300 | 0.268 | 0.252 | 0.521 | 0.079 |
| AcbC | 0.203 | 0.119 | 0.264 | 0.042 | | 0.067 | 0.020 | 0.110 | 0.073 | 0.081 | 0.052 | 0.063 | 0.016 | 0.220 | 0.070 | 0.031 |
| VMS | 0.002 | 0.303 | 0.516 | 0.003 | 0.067 | | 0.270 | 0.038 | 0.185 | 0.082 | 0.175 | 0.302 | 0.030 | 0.212 | 0.025 | 0.036 |
| DMS | 0.001 | 0.065 | 0.317 | 0.042 | 0.020 | 0.270 | | 0.000 | 0.562 | 0.539 | 0.818 | 0.745 | 0.058 | 0.592 | 0.129 | 0.070 |
| M1 | 0.020 | 0.055 | 0.604 | 0.022 | 0.110 | 0.038 | 0.000 | | 0.291 | 0.496 | 0.271 | 0.199 | 0.027 | 0.782 | 0.063 | 0.036 |
| V1 | 0.056 | 0.073 | 0.454 | 0.333 | 0.073 | 0.185 | 0.562 | 0.291 | | 0.660 | 0.459 | 0.561 | 0.622 | 0.870 | 0.803 | 0.648 |
| V1 | 0.068 | 0.140 | 0.563 | 0.269 | 0.081 | 0.082 | 0.539 | 0.496 | 0.660 | | 0.602 | 0.107 | 0.801 | 0.827 | 0.921 | 0.936 |
| DS | 0.059 | 0.086 | 0.388 | 0.355 | 0.052 | 0.175 | 0.818 | 0.271 | 0.459 | 0.602 | | 0.834 | 0.778 | 0.512 | 0.805 | 0.708 |
| DG | 0.097 | 0.105 | 0.330 | 0.300 | 0.063 | 0.302 | 0.745 | 0.199 | 0.561 | 0.107 | 0.834 | | 0.773 | 0.902 | 0.801 | 0.662 |
| PPx | 0.046 | 0.034 | 0.061 | 0.268 | 0.016 | 0.030 | 0.058 | 0.027 | 0.622 | 0.801 | 0.778 | 0.773 | | 0.375 | 0.704 | 0.707 |
| CA1 | 0.154 | 0.223 | 0.931 | 0.252 | 0.220 | 0.212 | 0.592 | 0.782 | 0.870 | 0.827 | 0.512 | 0.902 | 0.375 | | 0.700 | 0.836 |
| CA3 | 0.041 | 0.045 | 0.162 | 0.521 | 0.070 | 0.025 | 0.129 | 0.063 | 0.803 | 0.921 | 0.805 | 0.801 | 0.704 | 0.700 | | 0.493 |
| STN | 0.085 | 0.028 | 0.140 | 0.079 | 0.031 | 0.036 | 0.070 | 0.036 | 0.648 | 0.936 | 0.708 | 0.662 | 0.707 | 0.836 | 0.493 | |
| DLS | 0.339 | 0.039 | 0.067 | 0.059 | 0.044 | 0.045 | 0.010 | 0.026 | 0.050 | 0.075 | 0.070 | 0.045 | 0.001 | 0.530 | 0.019 | 0.011 |
| DLS | 0.073 | 0.072 | 0.069 | 0.026 | 0.051 | 0.068 | 0.307 | 0.106 | 0.158 | 0.064 | 0.085 | 0.061 | 0.002 | 0.534 | 0.050 | 0.028 |
| CeAMG | 0.064 | 0.064 | 0.825 | 0.009 | 0.113 | 0.156 | 0.455 | 0.431 | 0.379 | 0.369 | 0.326 | 0.503 | 0.031 | 0.853 | 0.099 | 0.113 |
| BlaAMG | 0.000 | 0.192 | 0.202 | 0.702 | 0.056 | 0.020 | 0.403 | 0.182 | 0.096 | 0.067 | 0.162 | 0.129 | 0.193 | 0.331 | 0.263 | 0.043 |
| A30c | 0.205 | 0.226 | 0.418 | 0.196 | 0.008 | 0.099 | 0.068 | 0.105 | 0.154 | 0.044 | 0.058 | 0.184 | 0.280 | 0.267 | 0.377 | 0.184 |
| A29c | 0.390 | 0.116 | 0.372 | 0.338 | 0.015 | 0.039 | 0.166 | 0.119 | 0.048 | 0.041 | 0.110 | 0.135 | 0.519 | 0.324 | 0.887 | 0.315 |
| MT | 0.176 | 0.139 | 0.555 | 0.248 | 0.048 | 0.162 | 0.586 | 0.112 | 0.887 | 0.439 | 0.786 | 0.737 | 0.102 | 0.592 | 0.138 | 0.204 |
| CT | 0.091 | 0.243 | 0.342 | 0.202 | 0.028 | 0.170 | 0.077 | 0.160 | 0.026 | 0.052 | 0.081 | 0.055 | 0.202 | 0.941 | 0.278 | 0.098 |
| OFC | 0.219 | 0.043 | 0.501 | 0.044 | 0.229 | 0.254 | 0.250 | 0.114 | 0.229 | 0.210 | 0.286 | 0.321 | 0.018 | 0.584 | 0.042 | 0.061 |
| IL | 0.252 | 0.102 | 0.109 | 0.029 | 0.130 | 0.166 | 0.030 | 0.131 | 0.065 | 0.081 | 0.055 | 0.062 | 0.067 | 0.186 | 0.052 | 0.045 |
| PrL | 0.003 | 0.287 | 0.296 | 0.133 | 0.235 | 0.033 | 0.091 | 0.020 | 0.048 | 0.072 | 0.051 | 0.068 | 0.080 | 0.127 | 0.055 | 0.064 |
| M2 | 0.105 | 0.031 | 0.092 | 0.348 | 0.100 | 0.151 | 0.064 | 0.158 | 0.137 | 0.133 | 0.306 | 0.209 | 0.127 | 0.429 | 0.162 | 0.062 |
| LOFC | 0.138 | 0.059 | 0.051 | 0.158 | 0.090 | 0.055 | 0.119 | 0.194 | 0.094 | 0.139 | 0.141 | 0.154 | 0.077 | 0.284 | 0.070 | 0.089 |
| Insula | 0.201 | 0.044 | 0.044 | 0.103 | 0.047 | 0.028 | 0.085 | 0.085 | 0.054 | 0.138 | 0.167 | 0.136 | 0.068 | 0.174 | 0.077 | 0.060 |
| LFC | 0.192 | 0.187 | 0.143 | 0.203 | 0.366 | 0.213 | 0.248 | 0.513 | 0.093 | 0.145 | 0.156 | 0.130 | 0.111 | 0.333 | 0.051 | 0.069 |
| ALM | 0.033 | 0.100 | 0.047 | 0.067 | 0.009 | 0.006 | 0.046 | 0.064 | 0.104 | 0.099 | 0.164 | 0.143 | 0.055 | 0.153 | 0.027 | 0.059 |

| | DLS | DLS | CeAMG | BlaAMG | A30c | A29c | MT | CT | OFC | IL | PrL | M2 | LOFC | Insula | LFC | ALM |
|---|---|---|---|---|---|---|---|---|---|---|---|---|---|---|---|---|
| AcbSh | 0.339 | 0.073 | 0.064 | 0.000 | 0.205 | 0.390 | 0.176 | 0.091 | 0.219 | 0.252 | 0.003 | 0.105 | 0.138 | 0.201 | 0.192 | 0.033 |
| A33 | 0.039 | 0.072 | 0.064 | 0.192 | 0.226 | 0.116 | 0.139 | 0.243 | 0.043 | 0.102 | 0.287 | 0.031 | 0.059 | 0.044 | 0.187 | 0.100 |
| A24a | 0.067 | 0.069 | 0.825 | 0.202 | 0.418 | 0.372 | 0.555 | 0.342 | 0.501 | 0.109 | 0.296 | 0.092 | 0.051 | 0.044 | 0.143 | 0.047 |
| A24b | 0.059 | 0.026 | 0.009 | 0.272 | 0.196 | 0.338 | 0.248 | 0.202 | 0.044 | 0.029 | 0.031 | 0.348 | 0.158 | 0.103 | 0.203 | 0.008 |
| AcbC | 0.044 | 0.051 | 0.113 | 0.056 | 0.008 | 0.015 | 0.048 | 0.028 | 0.229 | 0.130 | 0.235 | 0.100 | 0.090 | 0.047 | 0.366 | 0.009 |
| VMS | 0.045 | 0.068 | 0.156 | 0.020 | 0.099 | 0.039 | 0.162 | 0.170 | 0.254 | 0.166 | 0.033 | 0.151 | 0.055 | 0.028 | 0.213 | 0.006 |
| DMS | 0.010 | 0.307 | 0.455 | 0.403 | 0.068 | 0.166 | 0.586 | 0.077 | 0.250 | 0.030 | 0.091 | 0.064 | 0.119 | 0.085 | 0.248 | 0.046 |
| M1 | 0.026 | 0.106 | 0.431 | 0.182 | 0.105 | 0.119 | 0.112 | 0.160 | 0.114 | 0.131 | 0.020 | 0.158 | 0.194 | 0.085 | 0.513 | 0.064 |
| V1 | 0.050 | 0.158 | 0.379 | 0.096 | 0.154 | 0.048 | 0.887 | 0.026 | 0.229 | 0.065 | 0.048 | 0.137 | 0.094 | 0.054 | 0.093 | 0.104 |
| V1 | 0.075 | 0.064 | 0.369 | 0.067 | 0.044 | 0.041 | 0.439 | 0.052 | 0.210 | 0.081 | 0.072 | 0.133 | 0.139 | 0.138 | 0.145 | 0.099 |
| DS | 0.070 | 0.085 | 0.326 | 0.162 | 0.058 | 0.110 | 0.786 | 0.081 | 0.286 | 0.055 | 0.051 | 0.306 | 0.141 | 0.167 | 0.156 | 0.164 |
| DG | 0.045 | 0.061 | 0.503 | 0.129 | 0.184 | 0.135 | 0.737 | 0.055 | 0.321 | 0.062 | 0.068 | 0.209 | 0.154 | 0.136 | 0.130 | 0.143 |
| PPx | 0.001 | 0.002 | 0.031 | 0.193 | 0.280 | 0.519 | 0.102 | 0.202 | 0.018 | 0.067 | 0.080 | 0.127 | 0.077 | 0.068 | 0.111 | 0.055 |
| CA1 | 0.530 | 0.534 | 0.853 | 0.331 | 0.267 | 0.324 | 0.592 | 0.941 | 0.584 | 0.186 | 0.127 | 0.429 | 0.284 | 0.174 | 0.333 | 0.153 |
| CA3 | 0.019 | 0.050 | 0.099 | 0.263 | 0.377 | 0.887 | 0.138 | 0.278 | 0.042 | 0.052 | 0.055 | 0.162 | 0.070 | 0.077 | 0.051 | 0.027 |
| STN | 0.011 | 0.028 | 0.113 | 0.043 | 0.184 | 0.315 | 0.204 | 0.098 | 0.061 | 0.045 | 0.064 | 0.062 | 0.089 | 0.060 | 0.069 | 0.059 |
| DLS | | 0.258 | 0.047 | 0.303 | 0.101 | 0.187 | 0.082 | 0.217 | 0.173 | 0.591 | 0.325 | 0.102 | 0.468 | 0.271 | 0.594 | 0.043 |
| DLS | 0.258 | | 0.014 | 0.389 | 0.061 | 0.115 | 0.088 | 0.089 | 0.084 | 0.196 | 0.076 | 0.051 | 0.217 | 0.098 | 0.331 | 0.008 |
| CeAMG | 0.047 | 0.014 | | 0.166 | 0.040 | 0.103 | 0.369 | 0.033 | 0.532 | 0.052 | 0.038 | 0.007 | 0.043 | 0.014 | 0.016 | 0.002 |
| BlaAMG | 0.303 | 0.389 | 0.166 | | 0.022 | 0.361 | 0.076 | 0.076 | 0.068 | 0.153 | 0.020 | 0.732 | 0.315 | 0.431 | 0.365 | 0.004 |
| A30c | 0.101 | 0.061 | 0.040 | 0.022 | | 0.110 | 0.006 | 0.011 | 0.038 | 0.084 | 0.274 | 0.133 | 0.084 | 0.058 | 0.123 | 0.064 |
| A29c | 0.187 | 0.115 | 0.103 | 0.361 | 0.110 | | 0.001 | 0.020 | 0.071 | 0.073 | 0.189 | 0.284 | 0.081 | 0.102 | 0.083 | 0.071 |
| MT | 0.082 | 0.088 | 0.369 | 0.076 | 0.006 | 0.001 | | 0.008 | 0.412 | 0.120 | 0.259 | 0.254 | 0.113 | 0.105 | 0.189 | 0.143 |
| CT | 0.217 | 0.089 | 0.033 | 0.076 | 0.011 | 0.020 | 0.008 | | 0.047 | 0.224 | 0.395 | 0.251 | 0.099 | 0.056 | 0.214 | 0.185 |
| OFC | 0.173 | 0.084 | 0.532 | 0.068 | 0.038 | 0.071 | 0.412 | 0.047 | | 0.601 | 0.414 | 0.351 | 0.326 | 0.065 | 0.158 | 0.084 |
| IL | 0.591 | 0.196 | 0.052 | 0.153 | 0.084 | 0.073 | 0.120 | 0.224 | 0.601 | | 0.577 | 0.036 | 0.601 | 0.215 | 0.254 | 0.095 |
| PrL | 0.325 | 0.076 | 0.038 | 0.020 | 0.274 | 0.189 | 0.259 | 0.395 | 0.414 | 0.577 | | 0.097 | 0.449 | 0.196 | 0.335 | 0.003 |
| M2 | 0.102 | 0.051 | 0.007 | 0.732 | 0.133 | 0.284 | 0.254 | 0.251 | 0.351 | 0.036 | 0.097 | | 0.558 | 0.441 | 0.610 | 0.111 |
| LOFC | 0.468 | 0.217 | 0.043 | 0.315 | 0.084 | 0.081 | 0.113 | 0.099 | 0.326 | 0.601 | 0.449 | 0.558 | | 0.661 | 0.655 | 0.436 |
| Insula | 0.271 | 0.098 | 0.014 | 0.431 | 0.058 | 0.102 | 0.105 | 0.056 | 0.065 | 0.215 | 0.196 | 0.441 | 0.661 | | 0.387 | 0.184 |
| LFC | 0.594 | 0.331 | 0.016 | 0.365 | 0.123 | 0.083 | 0.189 | 0.214 | 0.158 | 0.254 | 0.335 | 0.610 | 0.655 | 0.387 | | 0.118 |
| ALM | 0.043 | 0.008 | 0.002 | 0.004 | 0.064 | 0.071 | 0.143 | 0.185 | 0.084 | 0.095 | 0.003 | 0.111 | 0.436 | 0.184 | 0.118 | |

**Supplementary 5. Quantity of p-values for analyzing the coherence between sham and TBI animals during periods of social.** M2, motor area 2; Prl, prelimbic cortex; IL, infralimbic cortex; VO, ventral orbitofrontal cortex; M1, motor area 1; LFC, lateral frontal cortex; AI, Anterior Insula; LO, Lateral orbitofrontal cortex; DMS, dorsomedial striatum; VMS, ventromedial striatum; AcbC, nucleus accumbens core; AcbSh, nucleus accumbens shell; MT, medial thalamus; CT, central thalamus; DLS, dorsolateral subiculum; CeAMG, central amygdala; BLaAMG, basolateral amygdala; STN, subthalamic nucleus; CA3, Cornu Ammonis 3 hippocampus; CA1, Cornu Ammonis 1 hippocampus; PPx, Posterior parietal cortex; DG, dentate g
